# Microbiota‐Derived Inosine Suppresses Systemic Autoimmunity via Restriction of B Cell Differentiation and Migration

**DOI:** 10.1002/advs.202409837

**Published:** 2025-04-28

**Authors:** Lingyu Gao, Yuhan Zhang, Zhi Hu, Shengwen Chen, Qiaolin Wang, Yong Zeng, Huiqi Yin, Junpeng Zhao, Yijing Zhan, Changxing Gao, Yue Xin, Bing Chen, Stijn van der Veen, Ming Zhao, Deyu Fang, Qianjin Lu

**Affiliations:** ^1^ Hospital for Skin Diseases Institute of Dermatology Chinese Academy of Medical Sciences and Peking Union Medical College Nanjing 210042 China; ^2^ Key Laboratory of Basic and Translational Research on Immune‐Mediated Skin Diseases Chinese Academy of Medical Sciences Nanjing 210042 China; ^3^ Jiangsu Key Laboratory of Molecular Biology for Skin Diseases and STIs Nanjing 210042 China; ^4^ Hunan Key Laboratory of Medical Epigenomics The Second Xiangya Hospital Central South University Changsha 410013 China; ^5^ Clinical Laboratory The Second Hospital of Anhui Medical University Hefei 230601 China; ^6^ Department of Microbiology and Department of Dermatology Sir Run Run Shaw Hospital School of Medicine Zhejiang University Hangzhou 310058 China; ^7^ Department of Pathology Northwestern University Feinberg School of Medicine Chicago IL 60611 USA

**Keywords:** systemic lupus erythematosus (SLE), lupus nephritis (LN), fecal microbiota transplantation (FMT), *Lactobacillus johnsonii*, purine metabolites, inosine, B cell differentiation, B cell migration

## Abstract

The role of gut microbiota dysbiosis in systemic lupus erythematosus (SLE) pathogenesis remains elusive. Here, it is shown that fecal microbiota transplantation (FMT) from healthy mice to lupus mice ameliorates lupus‐like symptoms. Microbiota reconstitution effectively reduces systemic class switch recombination (CSR) and elevates immunoglobulin heavy chain (IGH) naïve isotype. Microbiota profiling reveals an enrichment of *Lactobacillus johnsonii* post‐FMT, with a significant correlation to purine metabolites. Importantly, the *L. johnsonii*‐derived inosine, an intermediate metabolite in purine metabolism, effectively alleviates lupus pathogenesis in mice. Inosine inhibits B cell differentiation and reduces renal B cell infiltration to protect mice from lupus. At the molecular level, inosine reprograms B cells through the extracellular signal‐regulated kinase (ERK)‐hypoxia‐inducible factor‐1alpha (HIF‐1α) signaling pathway. Therefore, this study highlights the discovery of a novel microbial metabolite modulating autoimmunity and suggests its potential for innovative microbiome‐based therapeutic approaches.

## Graphical abstract

*Lactobacillus johnsonii* and its associated purine metabolite inosine not only restrict systemic B cell differentiation, but also contribute to immune cell infiltration in the kidneys, thereby serving dual functions in mitigating the progression of systemic lupus erythematosus (SLE). This study paves the way for innovative microbiome‐based therapeutic approaches.

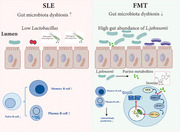

## 1 Introduction

Systemic lupus erythematosus (SLE) is a chronic and highly heterogeneous autoimmune disorder in which abnormal differentiation and activation of B cells, along with the secretion of autoantibodies, play central roles in its pathogenesis.^[^ ^1^ ^]^ During the maturation and expansion of B cells, there is a commencement of heightened antibody secretion, enhancing the efficacy of the adaptive immune response. In SLE, the identified autoantibodies often display high affinity and somatic mutations within IgG, indicating their formation in germinal centers where T cells contribute to the facilitation of class switching.^[^ ^2^ ^]^ Lupus nephritis (LN) is a type of glomerulonephritis and is one of the most severe organ manifestations associated with SLE. Renal damage in LN is orchestrated by autoreactive leukocytes, immune complexes, and various susceptibility genes related to SLE and LN. This pathological process is mediated by cytokines, chemokines, and growth factors.^[^ ^3^ ^]^

Gut microbiota is implicated in modulating metabolic networks and affecting systemic autoimmune pathogenesis.^[^ ^4^, ^5^, ^6^, ^7^, ^8^, ^9^ ^]^ Transplanting fecal material from SLE patients into germ‐free (GF) mice induced various lupus‐like phenotypic characteristics in the recipient GF mice.^[^ ^10^ ^]^ In addition, gut microbiota transplantation from MRL/lpr mice exacerbated the pathogenesis of pristane‐induced lupus mice and affected immune cell profiles in the intestine.^[^ ^11^ ^]^ SLE patients showed altered sulfur metabolism and flagella assembly.^[^ ^8^ ^]^ While triple congenic lupus‐prone mice exhibited aberrant tryptophan metabolism, exacerbating autoimmune phenotypes. Nevertheless, the impact of changes in gut microbiota on the progression of SLE remains to be fully elucidated.

Purine metabolites derived from the microbiota constitute a potent category of immune‐modulatory compounds capable of influencing the differentiation of regulatory T (Treg) cells, type 1 T helper (Th1) cells, and Th2 cells, as well as macrophages in both the gut and the peripheral regions.^[^ ^12^, ^13^, ^14^ ^]^ Probiotics *Lactobaccillus reuteri* altered the metabolomic profile and restored purine metabolite inosine, reducing Th1/Th2 cells. Conversely, in the presence of co‐stimulators such as dendritic cells (DCs) and the interleukin (IL)‐12 receptor, inosine derived from *Bifidobacterium pseudolongum* significantly promoted Th1 cells, thereby enhancing the effectiveness of checkpoint blockade immunotherapy. Importantly, both effects were contingent on activating the adenosine A2A receptor. However, how purine metabolites influence SLE remains elusive.

In this study, we found enrichment of *Lactobacillus johnsonii* and purine metabolites after effective fecal microbiota transplantation (FMT) treatment in lupus mouse models. *L. johnsonii* showed a strong correlation with purine metabolites. The intermediate metabolite inosine, the most apparent increased metabolite, ameliorated lupus‐like symptoms reducing systemic differentiated B cells and alleviating LN. Molecular inosine plays a pivotal role as a central intermediate in both purine biosynthetic and degradation pathways.^[^ ^15^ ^]^ Inosine inhibits B cell differentiation by triggering the ERK‐HIF‐1α signaling cascade. Monocolonized *L. johnsonii* led to a considerable rise in the concentration of the inosine. Collectively, our study identifies a specific FMT‐mediated gut bacterium and a microbiome‐derived metabolite that modulates autoimmunity and may be exploited to develop novel microbial‐based therapies.

## 2 Results

### 2.1 Lupus Mice Showed Gut Microbial Dysbiosis

To identify the potential changes of microbiota in lupus pathogenesis, we conducted 16S rDNA sequencing to dynamically analyze the microbial communities in fecal samples of MRL/lpr mice. Analysis of anti‐double‐stranded DNA (dsDNA) antibody level and circulating fluorescein isothiocyanate (FITC)–dextran confirmed the progression in lupus pathogenesis with aging in MRL/lpr mice (Figure S1A,B, Supporting Information). Importantly, a distinct bacterial translocation was observed in the mice kidney and mesenteric lymph nodes (MLNs) at 20 weeks of age, coinciding with the peak autoimmune response (Figure S1C, Supporting Information). Further 16S rDNA sequencing revealed dynamic variations in microbiome composition with aging, as evidenced by PCoA analysis (Figure S1D, Supporting Information), and there was a significant increase in alpha‐diversity in MRL/lpr mice at 10‐week (10w) old compared to the 4w mice (Figure S1E, Supporting Information), which were further confirmed at the phylum level (Figure S1F, Supporting Information). In contrast, the Firmicutes/Bacteroides ratio (F/B ratio) exhibited a notable decrease between 10w and 15w of age (Figure S1G, Supporting Information). Likewise, the *Firmicutes* phylum demonstrated a marked decline, but with a gradual increase in the abundance of the *Desulfobacterota* phylum in mice with aging (Figure S1G, Supporting Information). At the genus level, *Alistipes, Lactobacillus, Rikenella, and Clostridia UCG‐014* decreased, whereas *Bacteroides* increased, and *Desulfovibrio* were significantly reduced at 10w followed by a gradual increase thereafter (Figure S1H,I, Supporting Information). *Escherichia coli* showed a notable increase at the species level, especially at 20 weeks (Figure S1J, Supporting Information), whereas *L. johnsonii* and *L. murinus* showed marked decreases over time (Figure S1J, Supporting Information). Therefore, our data illustrated that a dynamic alteration in the microbiota composition accompanies the progression of autoimmunity in mice with lupus.

We then determined whether gut microbiota alterations contribute to autoimmune pathogenesis by treating MRL/lpr mice with a cocktail of antibiotics, which merely eliminated the entire intestinal flora (Figure S2A,B, Supporting Information). Antibiotics treatment decreased gut permeability (Figure S2C, Supporting Information) and reduced bacteria translocation into both the liver and kidney (Figure S2D, Supporting Information), along with ameliorating lupus‐like symptoms in mice, including a substantial reduction in urine protein levels (Figure S2E, Supporting Information) and the ratio of urine protein to creatinine (Figure S2F, Supporting Information). Although anti‐nuclear antibody (ANA) levels were unaltered (Figure S2H, Supporting Information), there was a marked reduction in plasma anti‐dsDNA antibodies by antibiotic treatment (Figure S2G, Supporting Information). Antibiotic treatment reduced the size and weight of the spleen (Figure S2I, Supporting Information). Additionally, both glomerulonephritis and immune cell infiltration in the kidney were alleviated, along with a reduced skin inflammatory response following antibiotic treatment (Figure S2J,K, Supporting Information). Flow cytometry analysis showed a dramatic decrease in CD4^+^ follicular helper T (Tfh) cells (Figure S2L, Supporting Information) with a significant increase in Foxp3^+^ Treg cells in antibiotic‐treated mice (Figure S2L, Supporting Information). Although with no obvious effect on memory B cells, germinal center B cells (GCB) and plasma cells, antibiotics treatment resulted in an increase in naïve B cells, but a reduction in plasmablasts in the spleen of antibiotic‐treated mice (Figure S2M, Supporting Information).

To rule out the possibility that the microbiota alterations are a strain‐specific event in MRL/lpr mice, we further utilized the pristane‐induced lupus mouse model using C57/B6 mice, which offers valuable insights into the role of type I interferon (IFN‐I), IFN‐Stimulated Genes and environmental triggers in the induction of lupus.^[^ ^16^, ^17^ ^]^ When compared to 20‐week (20w) old mice, the 40‐week (40w) old mice displayed significantly higher levels of urine protein and anti‐dsDNA antibodies, which are two key characteristics indicative of the onset of SLE (Figure S3A,B, Supporting Information). Lining up with the lupus disease progression, metagenome sequencing demonstrated distinct microbial communities in the feces of 20w and 40w mice by PCoA analysis (Figure S3C, Supporting Information). At the phylum level, there was a significant increase in the F/B ratio in 40w‐aged lupus mice (*p *< 0.001; Figure S3D, Supporting Information), which contrasts with the proportions observed in healthy individuals and SLE patients.^[^ ^18^ ^]^ We also observed a significant overrepresentation of the *Actinobacteria* (*p* < 0.0001) and *Verrucomicrobia* (*p *< 0.001) phyla in mice with onset lupus (Figure S3D, Supporting Information). At the genus level, genera such as *Alistipes*, *Bacteroides*, and *Prevotella* exhibited a significant decrease, while *Bifidobacterium*, *Faecalibaculum*, *Akkermansia*, and *Turicibacter* showed a significant increase (Figure S3E, Supporting Information). At the species level, dominantly increased species included *Bifidobacterium pseudolongum, Firmicutes bacterium M10‐2, Faecalibaculum rodentium, Akkermansia muciniphila*, and *L. reuteri* were the dominant increased species, whereas the relative abundance of *L. murinus* markedly decreased in mice with onset lupus (Figure S3F, Supporting Information). Collectively, our study demonstrated that the development of autoimmunity was concomitant with changes in microbial composition in both spontaneous and inducible lupus models, suggesting a critical contribution of microbiota dysbiosis in SLE pathogenesis and providing a rationale for correction of the microbial dysbiosis, such as by FMT, to prevent/treat the disease.

### 2.2 FMT Ameliorated Lupus‐Like Symptoms in Lupus Mouse Models

To select an optimal FMT donor, we conducted 16S rDNA sequencing on fecal microbiota samples from C56BL/6j (C57) and MRL/mpj (mpj) mice. C57 and mpj mice displayed distinct patterns of fecal microbial communities (Figure S4A, Supporting Information) without obvious alpha diversity differences (Figure S4B, Supporting Information). Compared to C57 mice, the dominant genus enriched in mpj mice is *Lactobacillus*, followed by increased *Rikenella* and decreased Clostridia_UCG‐014 (Figure S4C,D, Supporting Information). The genus *Lactobacillus* comprises various probiotics known to induce substantial alterations in host microbiota composition and to modulate the overall metabolic functions of intestinal microbiomes.^[^ ^19^ ^]^ Consequently, fecal microbiota from mpj mice were selected as the donor for FMT.

**Figure 1:**
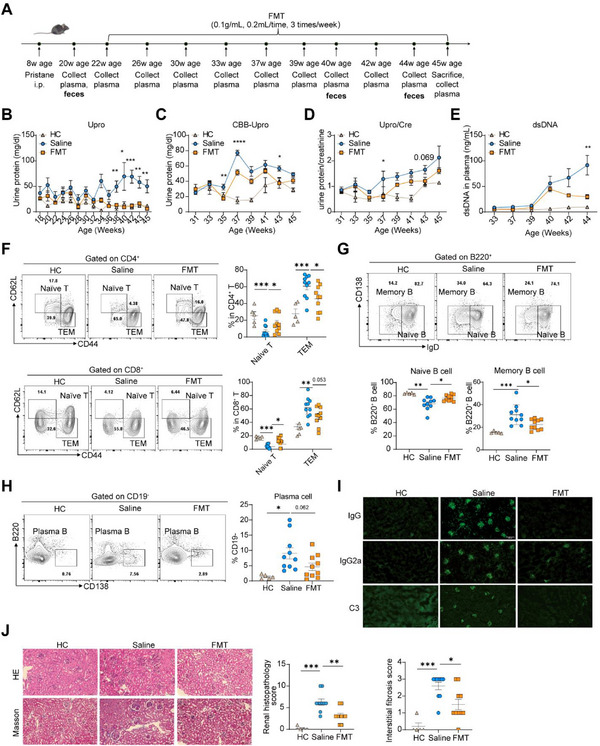
Transplantation of fecal microbiota from MRL/mpj mice attenuated lupus‐like phenotypes in pristane‐induced mice. A) Overview of FMT experimental design (*n* = 10 per group). 8 weeks age C57BL/6j mice were intraperitoneally injected 500 uL pristane, and lupus‐like symptoms started detecting at 18 weeks of age. FMT (0.1 g mL⁻^1^, 0.2 mL⁻^1^/time, 3 times/week) or saline treatment started at 22 weeks of age and lasted until 45 weeks of age. B,C) Urine protein was detected by B) test strip and C) CBB method. D) The ratio of urine protein to creatinine was detected. E) Plasma anti‐dsDNA level was detected between saline‐treated and FMT‐treated groups. F) Representative flow cytometry diagrams and statistical analyses were conducted to compare the frequencies of CD4 naïve T cells, CD4 TEM cells, CD8 naïve T cells, and CD8 TEM cells in the spleen between the saline‐treated and FMT‐treated groups. G) Representative flow cytometry diagrams and statistical analysis of frequencies of naive B cells and memory B cells in the spleen between saline‐treated and FMT‐treated groups. Renal histopathology was detected. Scale bar, 200 µm. H) Representative flow cytometry diagrams and statistical analysis of frequencies of plasma cells in the spleen between saline‐treated and FMT‐treated groups. I) IgG, IgG2a, and C3 deposition in the kidney were detected. Scale bar, 200µm. J) H&E staining and Masson staining of kidney, and renal histopathology score and interstitial fibrosis score were evaluated. Scale bar, 200 µm. The results are expressed as mean ± SEM. Statistical comparison was based on one‐way ANOVA. *p < 0.05 was considered statistically significant; **p < 0.01; ***p < 0.001; ****p < 0.0001.

The fecal microbiota from mpj mice were administered orally to mice with either pristane‐induced (C57) or spontaneous lupus (MRL/lpr) mice (**Figures** **1**A; S5A, Supporting Information). The FMT intervention continued for 24 weeks until a stable alleviation of lupus‐like symptoms was observed in pristane‐induced lupus mice (Figure 1A). Remarkably, FMT dramatically reduced the levels of urine protein (Figure 1B,C; Figure S5B, Supporting Information), the urine protein‐to‐creatinine ratio (Figure 1D), and the anti‐dsDNA antibody levels in lupus mouse models (Figure 1E). A similar lupus‐protective efficacy was observed by a 6‐weeks of FMT treatment in MRL/lpr mice (Figure S5C,D, Supporting Information). Flow cytometry analysis detected a dramatic decrease in the percentages of both effective memory T (CD4^+^ and CD8^+^) and B effector memory cells by the FMT‐treatment, with a concurrent increase in the frequency of their naïve cells (Figure 1F,G). In addition, we observed a noticeable reduction in plasma cells (*p* = 0.062, Figure 1H), and lower levels of IgG, IgG2a, and C3 deposition in kidneys compared to those in saline‐treated mice (Figure 1I; Figure S5E, Supporting Information). Histological analysis revealed that FMT intervention reduced immune cell infiltration, ameliorated glomerulonephritis, and decreased interstitial fibrosis (Figure 1J; Figure S5E, Supporting Information). In MRL/lpr mice, FMT treatment resulted in a significant decrease in the frequency of Tfh cells (*p* < 0.05) and Th1 cells (*p* <0.01) (Figure S5F, Supporting Information). In summary, via modulating autoimmunity, FMT effectively alleviated lupus‐like symptoms in both pristane‐induced and spontaneous lupus mice.

### 2.3 FMT Reduced Class‐Switch Recombination and Increased IGH Naïve Isotype in Lupus Mice

**Figure 2:**
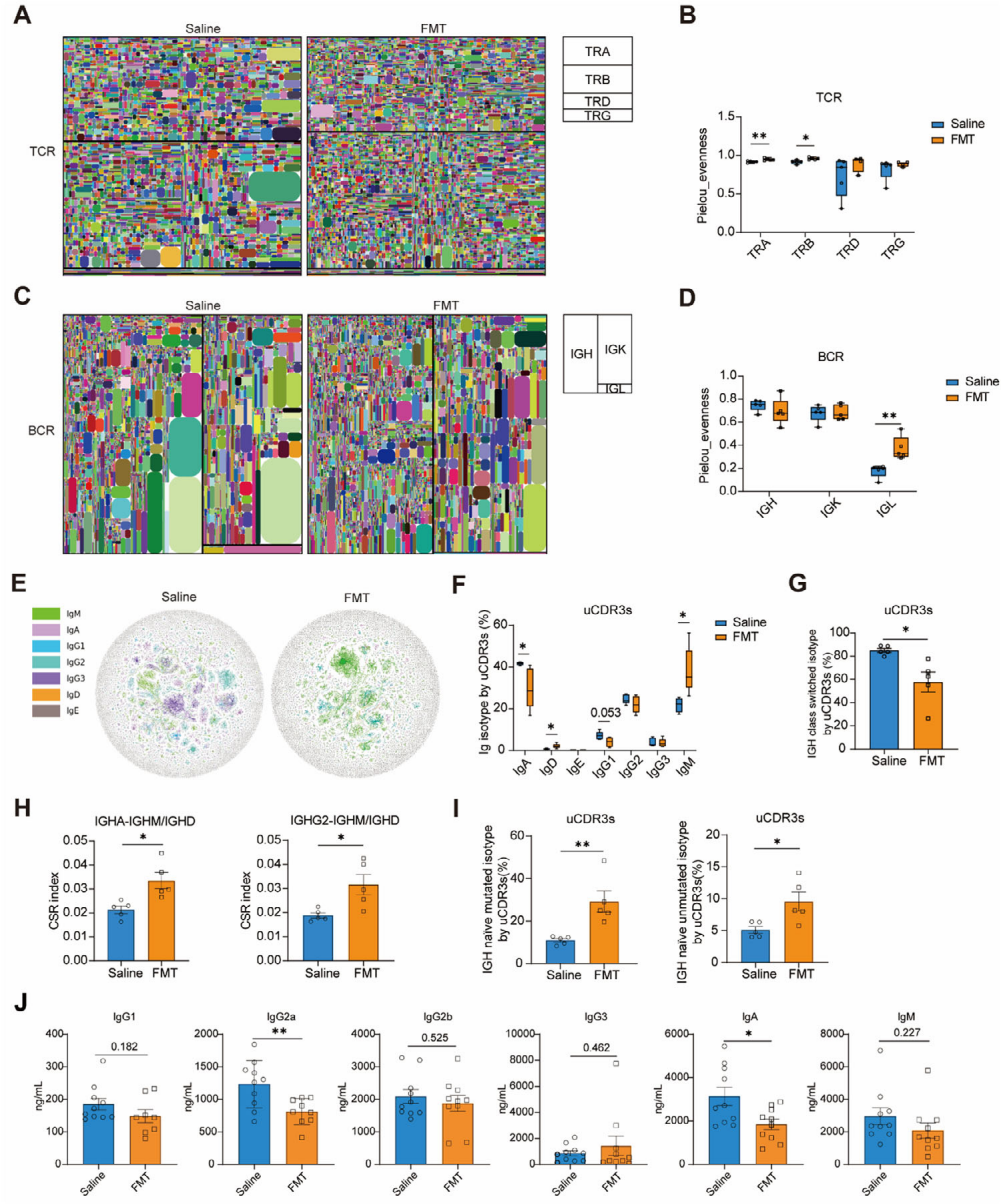
FMT reduced CSR and increased IGH naïve isotype in lupus mice. A) Tree maps of the TCR IR of group saline and FMT. Each uCDR3 clone is treated as a pane with different colors, and the size of the panes is based on expression. Panes were gathered from top to bottom as TRA, TRB, TRD, and TRG. B) The Pielou evenness of TRA, TRB, TRD, and TRG chains. C) Tree maps of the BCR IR of group saline and FMT. Panes were gathered from top to bottom and from left to right as IGH, IGK, and IGL. D) The Pielou evenness of IGH, IGK, and IGL chains. E) Networks of IGHs from group saline and FMT. Each node represents a uCDR3 sequence based on expression, and the sequences that underwent CSR or SHM are linked by sticks. F) Expression percentage calculated by uCDR3s in each IGH chain isotype from group saline and FMT. G) IGH class switched isotype percent by uCDR3s. H) The CSR index of IGHA and IGHG2 with IGHM/IGHD (IGHM and IGHD are shown in the graph as a whole) in the two groups. I) IGH naïve mutated isotype percentage and IGH naïve unmutated isotype percentage by uCDR3s. J) The plasma levels of total IgG1, IgG2a, IgG2b, IgG3, IgA, and IgM between saline‐treated and FMT‐treated group. The results are expressed as mean ± SEM. Statistical comparison was based on an unpaired Student *t*‐test. *p < 0.05 was considered statistically significant; **p < 0.01.

One critical question is whether gut microbiota transplantation influenced autoimmunity, in addition to impacting on T cells, B cells, and ig isotypes, and altering the repertoire of T cell receptors (TCR) and B cell receptors (BCR). We then performed an immune repertoire (IR) sequencing encompassing all seven TCR/BCR chains, including TCR alpha (TRA), TCR beta (TRB), TCR delta (TRD), TCR gamma (TRG), BCR heavy (IGH), BCR kappa (IGK), and BCR lambda (IGL), allowing for a comprehensive analysis of the composition of BCR and TCR (**Table** **1**). The total TCR and BCR expression levels showed no apparent IR alterations between FMT and control groups (Figure S6A, Supporting Information). While the mean length of complementarity‐determining regions (CDR3s) for all four chains in TCR, as well as IGK and IGL in BCR, remained unchanged by FMT treatment, a shorter mean CDR3 length was detected in IGH of BCR (Figure S6B, Supporting Information). Here, we presented repertoire tree maps depicting the four chains of TCR (**Figure** **2**A) and the three chains of BCR (Figure 2C). The Pielou evenness analysis revealed that FMT treatment led to an increase in TRA and TRB diversity (Figure 2B) and elevated IGL diversity when compared with saline‐treated lupus mice (Figure 2D). To visualize the intricate somatic hypermutation (SHM) and CSR events within IGH clones of both the saline and FMT groups, we created network diagrams illustrating all IGH clones from a representative lupus mouse (Figure 2E). Each colored circle in the diagram represents a specific IGH clone, with the size of circle corresponding to its expression level. Clones connected by short lines indicate that they differ by only one amino acid in their primary structure. The network diagrams vividly depict the complex SHM and CSR processes within the IGH repertoires of saline‐treated pristane‐induced mice. In contrast, FMT treatment appeared to reduce SHM and CSR, as fewer linked connections between nodes were observed (Figure 2E). Quantification at the reading level showed that, in contrast to the fact that the IgA and IgG2 were the predominant Ig isotypes in saline‐treated pristane‐induced mice, FMT treatment led to a significant increase in IgM and IgD (Figure S6C, Supporting Information). At the unique CDR3s (uCDR3s) level, FMT treatment resulted in a significant decrease in the percentage of IgA and a slight decrease in IgG1, while there was a significant increase in IgD and IgM in the FMT‐treated mice (Figure 2F), which are consistent with our discovery that FMT increased the naïve B cell frequency (Figure 1G). In addition, regarding the SHM frequency among the BCR Ig isotypes (Figure S6D, Supporting Information), FMT treatment led to a reduction in the percentage of switched IGH class isotypes, as indicated by both read counts and uCDR3s level (Figure 2G; Figure S6C, Supporting Information). Furthermore, FMT significantly increased the CSR index of IGHA with IGHM/IGHD and IGHG2 with IGHM/IGHD (Figure 2H), but decreased the CSR index of IGHA with IGHG1, IGHG1 with IGHG2, and IGHG1 with IGHG3 (Figure S6E, Supporting Information). Then, we calculated the percentage of naïve mutated and unmutated isotypes within IGH using read counts and uCDR3, and FMT treatment led to an increase in all these percentages (Figure 2I; Figure S6F, Supporting Information). We also found that the FMT treatment notably attenuated the secretion of IgG2a and IgA (Figure 2J). In summary, FMT restricted T and B cell activation and maintained a higher frequency of naïve phenotype. At molecular levels, FMT increased TCR diversity and the percentages of IgD and IgM, but reduced IgA levels.

**Table 1:** Overall analysis of the composition of BCR and TCR.

Sample Name	Organ	TCR				BCR			
		Decoded Reads	High Quality Reads	Reads/UMI	Total Receptor RNA	Decoded Reads	High Quality Reads	Reads/UMI	Total Receptor RNA
Saline‐1	Spleen	3 323 368	3 098 950	135.65	18 426	2 192 790	1 980 867	7.79	198 434
Saline‐2	Spleen	3 992 458	3 727 833	271.04	10 811	2 493 495	2 240 138	25.06	81 403
Saline‐3	Spleen	3 777 215	3 546 120	263.81	10 599	2 203 469	1 967 004	20.31	81 534
Saline‐4	Spleen	3 963 596	3 678 978	81.64	36 265	2 082 622	1 876 413	5.63	283 177
Saline‐5	Spleen	3 625 882	3 366 833	90.29	30 810	2 405 557	2 154 355	6.19	261 238
FMT‐1	Spleen	3 600 634	3 358 532	66.80	40 258	2 314 091	2 091 381	4.06	381 823
FMT‐2	Spleen	2 672 066	2 438 569	1 539.50	190	2 578 700	2 301 964	50.34	33 110
FMT‐3	Spleen	3 376 089	3 148 216	59.41	43 166	2 433 317	2 173 678	5.32	293 634
FMT‐4	Spleen	4 075 064	3 815 009	190.86	16 505	2 079 947	1 879 126	24.95	66 224
FMT‐5	Spleen	4 475 242	4 143 323	70.20	48 561	2 337 856	2 095 932	4.48	383 390

TCR, t cell receptor; BCR, b cell receptor; FMT, fecal microbiota transplantation

### 2.4 FMT Reduced Immune Cell Infiltration in Kidney of Lupus Mice

**Figure 3:**
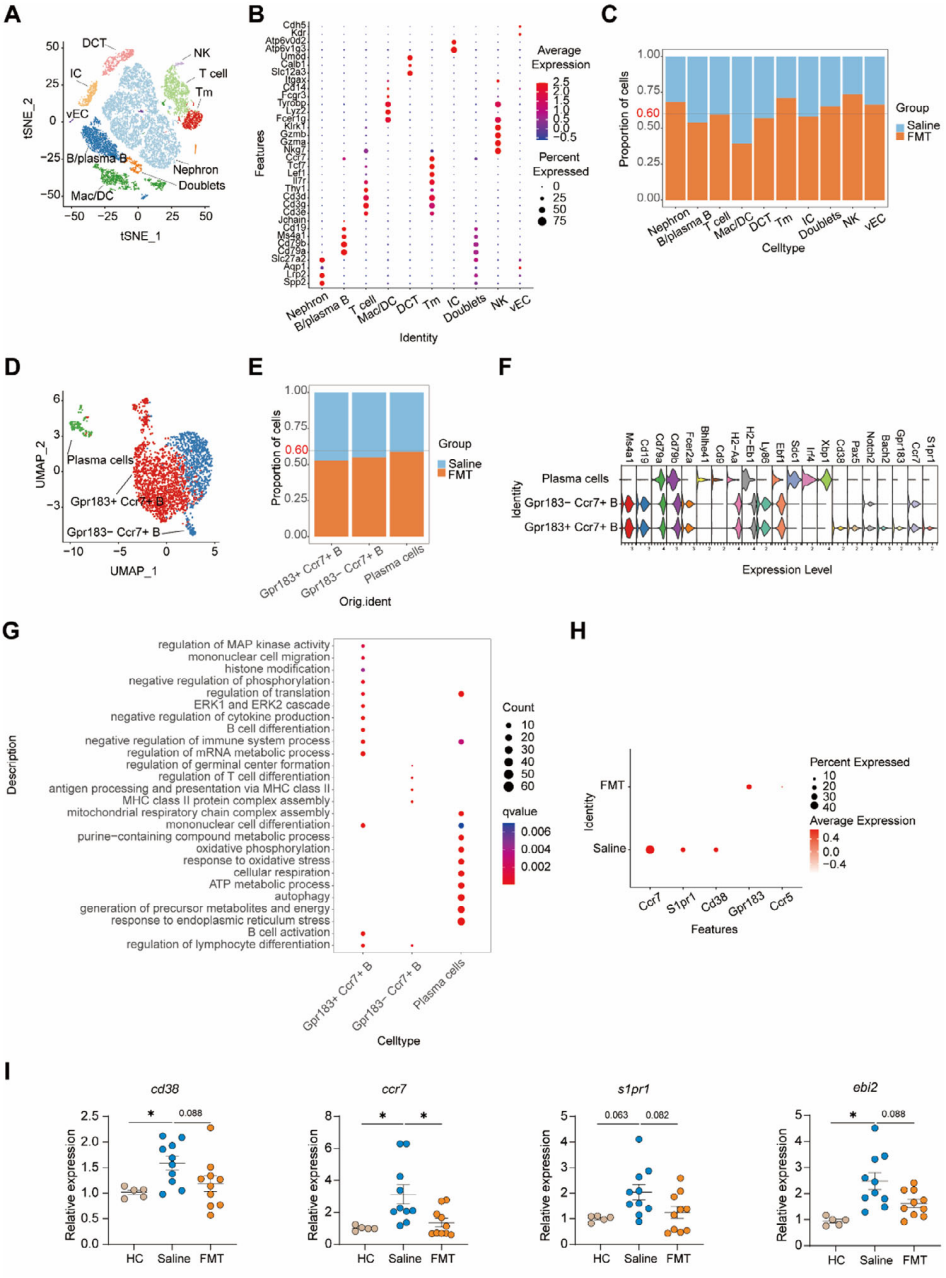
Gut microbiota alteration reduced immune cell infiltration in mice kidneys. A) scRNA‐seq t‐distributed stochastic neighbor embedding (t‐SNE) plot exhibiting renal cells from FMT‐treated pristane‐induced mice (FMT, *n* = 3) and saline‐treated controls (Saline, *n* = 2). Cells colored by cell types. Macrophages and dendritic cells: Mac/DC, distal convoluted tubule: DCT, memory T cell: Tm, intercalated cell: IC, vascular endothelial cell: vEC. B) Dot plot shows signature genes for each cell type. The dot color represents the scaled amount of expression for each gene. The size of the dot represents the percentage of cells expressing the gene. C) Bar plot showing the proportion of cells from different groups in each cell type. D) Umap plot exhibiting individual renal B cells colored by sub‐clusters. E) A bar plot shows the proportion of cells from different groups in each sub‐cluster of B cell. F) Violin plot showing the expression level of signature genes for each sub‐cluster of B cells. Colors represent different genes. G) Dot plot visualizes the GO biological process terms significantly enriched in each sub‐cluster of B cells from saline and FMT. H) Dot plot showing genes of interest for each cell type. The dot color represents the scaled amount of expression for each gene. The size of the dot represents the percentage of cells expressing the gene. I) The mRNA level of *cd38, ccr7, s1pr1*, and *ebi2* in kidney. The results are expressed as mean ± SEM. Statistical comparison was based on one‐way ANOVA. *p < 0.05 was considered statistically significant; **p < 0.01; ***p < 0.001; ****p < 0.0001.

To characterize the heterogeneity of intra‐renal cell subsets involved in FMT‐induced renoprotection, we performed single‐cell RNA sequencing (scRNA‐seq) to profile 19695 renal cells from kidney samples dissected from FMT‐treated lupus mice and saline‐treated controls (**Figure** **3**A; Figure S7A, Supporting Information). After mapping the cluster marker genes to established cell type‐defining signature genes, we identified ten cell types including nephron (*Slc27a2, Lrp1, Spp1*), B cell (B/plasma B; *Cd79a, Cd79b, Ms4a1, Jchain*), T cell (*Cd3e, Cd3d, Thy1*), macrophages and dendritic cells (Mac/DC; *Lyz2, Tyrobp, Itgax*), distal convoluted tubule (DCT; *Slc12a3, Calb1, Umod*), memory T cell (Tm; *Tcf7, Lef7, Ccr7*), intercalated cell (IC; *Atp6v0d2, Atp6v1g3*), vascular endothelial cell (vEC; *Cdh5, Kdr*), and doublets exhibiting both B cell and nephron signature genes (Figure 3B; Figure S7B, Supporting Information).^[^ ^20^, ^21^, ^22^ ^]^ The analysis of cell composition in different cell types exhibited a reduction in B/plasma B and Mac/DC after FMT (Figure 3C; Figure S7C, Supporting Information). The changes in cell proportions were further confirmed in mice kidneys with markers of key cell types: B cell (*Cd19*), macrophages (*F4/80*), DC (*Cd11c*) (Figure S7D–F, Supporting Information). B cells and macrophages consistently reduced after FMT treatment (Figure S7D–F, Supporting Information). Furthermore, FMT dramatically changed gene expression in cell clusters, with inflammatory‐related genes downregulated, including Cxcr4, Jak1 in B cell cluster (Figure S7H, Supporting Information); *Ly6e*, Cxcr3, Il7r in T cell cluster (Figure S7I, Supporting Information); Ly6c in Tm cluster (Figure S7J, Supporting Information); S100a4, Cxcr4, Itga4, Ly6i, Il6ar in macrophages (Figure S7K, Supporting Information). These results indicate that FMT inhibits the inflammatory response in T, B cells and macrophages in the kidney of mice.

It has been shown that B cell infiltration in human kidney biopsies correlates with lupus disease severity as documented by the heightened levels of creatinine, blood urea nitrogen, and urine protein.^[^ ^23^, ^24^, ^25^, ^26^ ^]^ Indeed, based on the expression of Gpr183 and Ccr7, which implicates B cell migration,^[^ ^27^, ^28^, ^29^ ^]^ we identified the Gpr183^low^ Ccr7^high^ and Gpr183^high^ Ccr7^high^ B cell clusters (Figure 3D; Figure S7G, Supporting Information), both of which showed a decreased in cell proportion after FMT (Figure 3E). Plasma cells were clustered and annotated by canonical markers including *Cd79a, Cd79b, Sdc1, Irf4*, and *Xbp1* (Figure 3D,F).^[^ ^30^, ^31^ ^]^ We noticed that Gpr183^high^ ccr7^high^ B cell highly expressed *Cd38, Pax5* and *Bach2*, which are feature genes of mature memory B cell (Figure 3F). We also noticed that this cell cluster expressed a high level of *S1pr1*, which implied a high migration ability (Figure 3F). Gene Ontology (GO) analysis of differential genes changes in Gpr183^high^ Ccr7^high^ B cell cluster revealed FMT modulated mitogen‐activated protein kinases (MAPK) activity, mononuclear cell migration, histone modification and cell differentiation (Figure 3G); while Gpr183^low^ Cc7^high^ B cell cluster was associated with regulation of germinal center formation, T cell differentiation and antigen processing and presentation (Figure 3G). Furthermore, we identified that FMT down‐regulated the total expression level of *Ccr7, S1pr1*, and *Cd38*, while upregulating *Gpr183* and *Ccr5* (Figure 3H).

We further verified the effect of FMT on B cell differentiation and migration to kidney by analyzing the *Cd38*, *Ccr7*, *S1pr1* and *Gpr183* expression. Consistently, FMT markedly reduced the mRNA expression of *Ccr7* and exhibited a decreasing trend in the mRNA levels of *Cd38*, *S1pr1*, and *Ebi2* in the kidney (Figure 3I). In summary, we discovered that the predominant B cell population in pristane‐induced mice consists of Ccr7^high^ B cells.

### 2.5 FMT Modulated Gut Microbiome Homeostasis and Increased *Lactobacillus*

**Figure 4:**
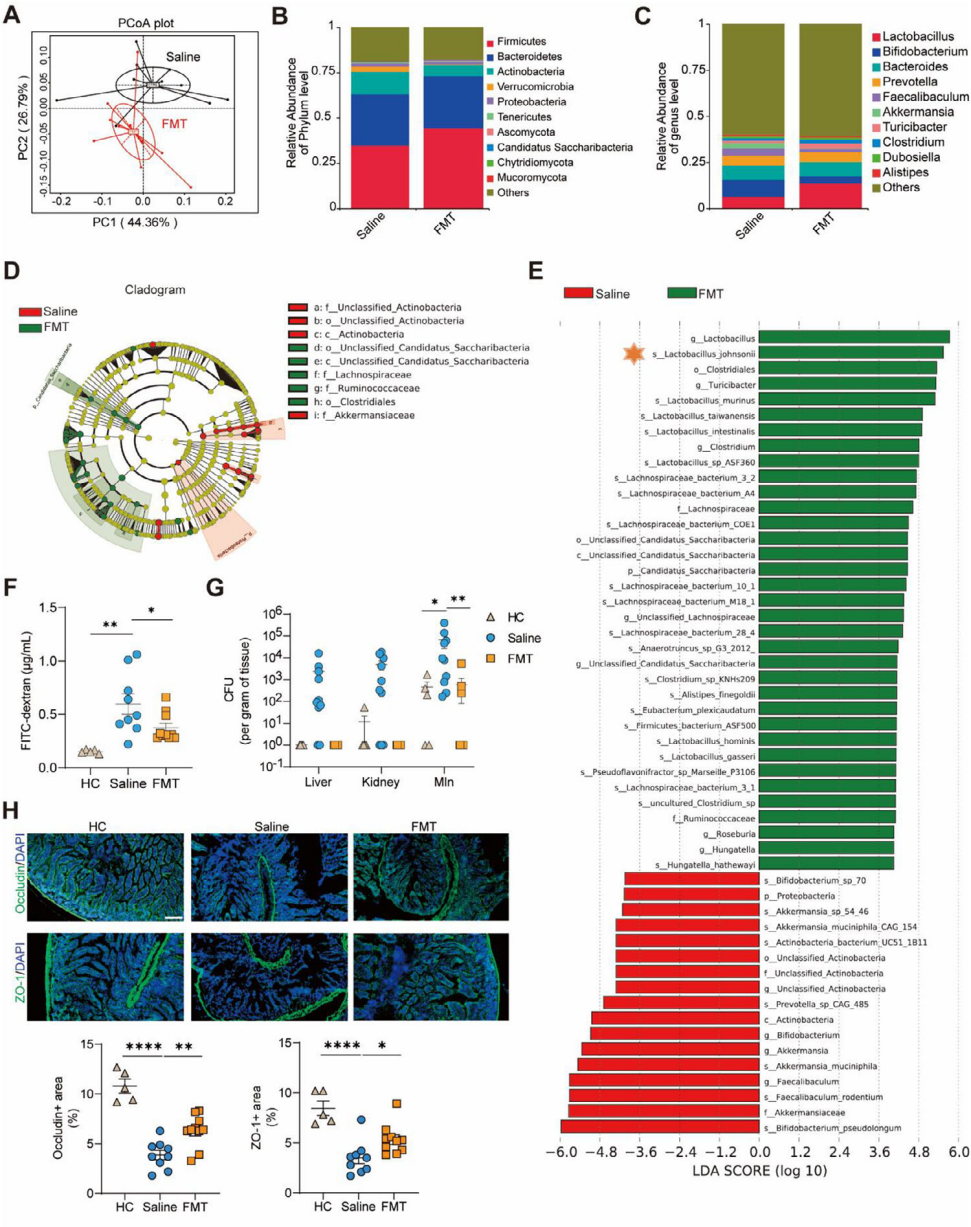
FMT modulated gut microbiome in pristane‐induced mice. Metagenome sequencing analysis of DNA from fecal pellets in saline‐treated and FMT‐treated pristane‐induced mice (*n* = 10 per group) in 44 weeks. A) Beta‐diversity using principal coordinate analysis of Bray‐Curtis was performed between groups. B) Relative abundance of the top 10 phyla in saline‐treated and FMT‐treated groups. C) Relative abundance of the top 10 genera in saline‐treated and FMT‐treated groups. D) The cladogram generated using LEfSe shows statistically significant differences between saline‐ and FMT‐treated groups. E) A Histogram of the LDA scores generated with LEfSe is shown on a logarithmic scale. The most differential taxa are displayed in the FMT group in green and the saline group in red. F) Plasma FITC‐dextran level in 45 weeks of age among healthy control (HC), saline‐treated and FMT‐treated groups. G) Cultures of translocated bacteria in liver, kidney, and MLN among HC, saline‐treated and FMT‐treated groups. H) Immunofluorescence of tight junction Occludin and ZO‐1 were detected in the small intestines. The results are expressed as mean ± SEM. Statistical comparison was based on one‐way ANOVA in (F). *p < 0.05 was considered statistically significant; **p < 0.01; ***p < 0.001.

Subsequently, we delved into analyzing the possible microbiota alterations by FMT that contribute to autoimmune inhibition in lupus mice. Metagenome sequencing revealed that FMT significantly influenced microbial diversity and composition (**Figure** **4**A–C). PCoA indicated a significant distinction in the spread or distribution of data points between the groups (Figure 4A). At the phylum level, there was no significant changes in the F/B ratio (Figure S8A, Supporting Information), while Phylum *Actinobacteria* (*p* < 0.01) and *Verrucomicrobia* (*p* < 0.001) exhibited a significant decrease following FMT treatment (Figure S8A, Supporting Information). At the genus level, *Lactobacillus* showed a marked increase (*p *< 0.001), while *Bifidobacterium* (*p* < 0.01) and *Akkermansia* (*p* < 0.0001) exhibited significant decreases post‐FMT treatment (Figure S8B, Supporting Information). This treatment effectively modulated the altered microbial composition associated with lupus pathogenesis (Figure S3E, Supporting Information). A cladogram vividly illustrated the evolutionary and phylogenetic relationships among microbial communities followed FMT treatment (Figure 4D). Marker clusters were found to be enriched with FMT treatment, including *Candidatus Saccharibacteria, Lachnospiraceae, Rumonococcaceae, and Clostridiales* (Figure 4D). Linear discriminant analysis (LDA) of effect size, used to elucidate the key microbial taxa that contributing the variation of FMT‐treated gut microbiota, indicated that *L. johnsonii* emerged as the dominant species in mice after FMT (Figure 4E). Additionally, FMT treatment effectively prevented gut leakiness in the pristane‐induced lupus model (Figure 4F). The bacterial translocation to the liver, MLN, and kidney was largely blocked by FMT in pristane‐induced mice (Figure 4G). Additionally, FMT elevated the expression of tight junction proteins, Occludin and Zo‐1 (Figure 4H). We also observed less immune cell infiltration in small intestines and colonic villi undergoing an increase in height with FMT treatment (Figure S9A,B, Supporting Information). In summary, FMT modulated gut microbiota structures by elevating *L. johnsonii* and improved microbiome homeostasis with improved gut barrier integrity and reduced bacterial translocation to distant organs.

### 2.6 *L*. *Johnsonii*‐Associated Purine Metabolites Were Enriched by FMT to Alleviate Systemic Autoimmunity

**Figure 5:**
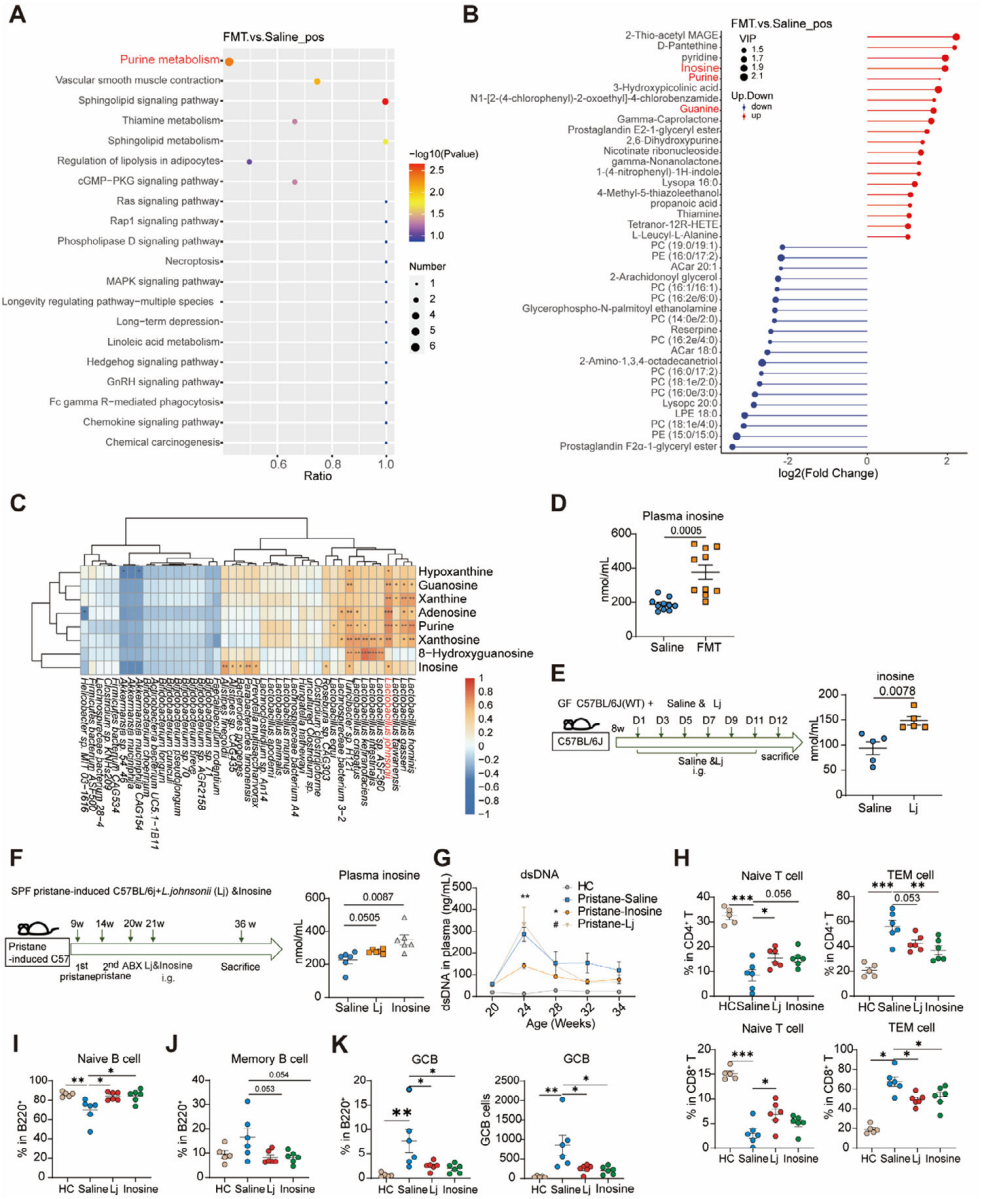
*L.johnsonii*‐associated purine metabolite enrichment alleviated systemic autoimmunity. An untargeted metabolomics of the feces in saline‐treated and FMT treated pristane‐induced mice in 44 weeks was conducted (*n* = 10 per group). A) Enriched KEGG pathways in fecal metabolites between saline‐treated and FMT‐treated mice were shown with the P values and the number of metabolites represented in each pathway. The size of each bubble represents the number of metabolites differentially expressed for each pathway, with a scale on the lower right (number). B) The matchstick map according to the differential metabolites obtained from each group of difference comparison combinations in positive mode was drawn, and the up‐down of metabolites and the substances with large difference multiples were indicated. C) Pearson's correlation analysis between metabolites in purine metabolism and significantly differentiated strains was conducted. D) Plasma inosine level was tested between Saline‐treated and FMT‐treated groups. E) Schematic diagram of GF mice orally treated saline and *L. johnsonii* (Lj, 2 OD, 0.3 mL⁻^1^/time, 6 times in total). Plasma inosine level was tested between saline‐treated and Lj‐treated groups. F) Overview of Lj and inosine treatment experimental design (*n* = 6 per group) in pristane‐induced C57 mice. Orally gavage Lj (2 OD, 0.3 mL⁻^1^/time, 3 times/week), inosine (50 mg k^−1^g, 0.3 mL⁻^1^/time, 3 times/week), or saline started at 21 weeks of age, and lasted until 36 weeks of age. The amount of inosine in plasma from saline‐treated, Lj‐treated and inosine‐treated pristane‐induced mice was detected. G) Plasma anti‐dsDNA level was detected among HC, saline‐treated, Lj‐treated, and inosine‐treated groups. H) Statistical analyses were conducted to compare the systemic frequencies of CD4 naïve T cells and CD4 TEM cells, CD8 naïve T cells and CD8 TEM cells among HC, saline‐treated, Lj‐treated, and inosine‐treated groups. I,J) Statistical analyses were conducted to compare the systemic frequencies of I) naïve B and J) memory B cells among HC, saline‐treated, Lj‐treated, and inosine‐treated groups. K) Statistical analyses were conducted to compare the systemic frequencies and cell number of GCB among saline‐treated, Lj‐treated, and inosine‐treated groups. The results are expressed as mean ± SEM. Statistical comparison was based on Student *t*‐test and a one‐way ANOVA. *p < 0.05 was considered statistically significant. **p < 0.01; ***p < 0.001.

To uncover the mechanisms underlying how gut microbiota alteration alleviates autoimmunity, we conducted untargeted metabolic profiling of fecal samples from FMT‐treated and saline‐treated pristane‐induced lupus mice. Indeed, a distinct separation was detected in fecal metabolites between the saline‐treated and FMT‐treated pristane‐induced mice (Figure S10A,B, Supporting Information). A total of 883 metabolites in the positive ion mode, and 531 metabolites in the negative ion mode were identified, with 150 metabolites and 84 metabolites being dramatically altered by FMT. Out of the 150 metabolites in the positive ion mode significantly altered by FMT, 67 metabolites were up‐regulated, and 83 metabolites were down‐regulated. On the other hand, 84 metabolites in the negative ion mode showed significant differences, with 31 metabolites being up‐regulated and 53 metabolites being down‐regulated (Extended Table 1 and **Table** **2**). Further KEGG enrichment analysis, performed for positive ion modes, consistently highlighted purine metabolism as the predominant enriched pathway among the differential metabolites (**Figure** **5**A). The matchstick map was generated based on the differential metabolites identified from each comparison group, providing a clear visualization of the metabolite changes and substances with significant differences in abundance (Figure 5B; Figure S10C, Supporting Information). Importantly, a dramatic increase in fecal inosine and purine levels in the positive ion mode, along with elevated levels of xanthosine and guanosine in the negative ion mode was detected in FMT‐treated mice (Figure 5B; Figure S10C, Supporting Information). Of note, inosine, an intermediate product in purine biosynthesis and a secondary metabolite of purine degradation (Figure S10D, Supporting Information), exhibited a 3.85‐fold increase in FMT versus control mice (Figure S10E, Supporting Information).

**Table 2:** Key resources table.

REAGENT or RESOURCE	SOURCE	IDENTIFIER
**Antibodies**		
Brilliant Violet 421™ anti‐mouse CD138 (Syndecan‐1) Antibody	Biolegend	142508
PerCP/Cyanine5.5 anti‐mouse IgD Antibody	Biolegend	405710
PE Rat Anti‐Mouse CD19	Biolegend	152408
PE/Cyanine7 anti‐mouse CD62L Antibody	Biolegend	104418
PE‐CF594 anti‐mouse CD95 (Fas) Antibody	BD	562499
FITC anti‐mouse CD3 Antibody	Biolegend	100204
Alexa Fluor® 700 anti‐mouse CD4 Antibody	BD	557956
APC‐eF780 Rat Anti‐Mouse CD8a	Invitrogen	47‐0081‐82
BV605 anti‐mouse/human CD45R/B220 Antibody	Biolegend	103244
Alexa Fluor® 647 anti‐mouse/human GL7 Antigen	Biolegend	144606
mouse Fc‐R block	Biolegend	101302
Brilliant Violet 785™ anti‐mouse/human CD44 Antibody	Biolegend	103059
FITC anti‐mouse/human CD44 Antibody	Biolegend	103022
Zombie NIR™ Fixable Viability Kit	Biolegend	423106
Brilliant Violet 785™ anti‐mouse CD197 (CCR7) Antibody	Biolegend	120127
Brilliant Violet 785™ anti‐mouse CD25 Antibody	Biolegend	102051
BD Pharmingen™ Biotin Rat Anti‐Mouse CD185 (CXCR5)	BD	551960
BD Horizon™ BB700 Hamster Anti‐Mouse CD279 (PD‐1)	BD	566514
APC Streptavidin	Biolegend	405207
PE‐CF594 Rat Anti‐Mouse IFN‐γ	BD	562303
BD Horizon™ BV421 Rat Anti‐Mouse IL‐17A	BD	563354
PE/Cyanine7 anti‐mouse IL‐4 Antibody	Biolegend	504118
BD Pharmingen™ PE Rat anti‐Mouse Foxp3	BD	563101
BD Horizon™ BV650 Mouse Anti‐Human CD19	BD	563226
BD Pharmingen™ PerCP‐Cy™5.5 Mouse Anti‐Human CD38	BD	551400
BD Pharmingen™ PE‐Cy™7 Mouse Anti‐Human CD69	BD	557745
BD Pharmingen™ APC Mouse Anti‐Human IgD	BD	561303
BD Pharmingen™ FITC Mouse Anti‐Human CD20	BD	556632
PE/Dazzle™ 594 anti‐mouse/rat/human CD27 Antibody	Biolegend	124228
Goat Anti‐Rabbit IgG (FITC)	Proteintech	SA00003‐2
Goat Anti‐Mouse IgG2a heavy chain (FITC)	Abcam	ab97244
C3/C3b/C3c Polyclonal antibody	Proteintech	21337‐1‐AP
HRP Goat Anti‐Mouse IgG1	Abclonal	AS066
HRP Goat Anti‐Mouse IgG2a	Abclonal	AS065
IgG2b heavy chain (HRP)	Abcam	ab97250
Goat anti‐Mouse IgG3 Secondary Antibody, HRP	Invitrogen	M32607
HIF‐1α (D1S7 W) XP® Rabbit mAb	CST	36169
ERK1/2 Antibody	AFFINITY	AF0155
Phospho‐ERK1/2 (Thr202/Tyr204) Antibody	AFFINITY	AF1015
AKT1 Rabbit mAb	Abclonal	WH385740
Phospho‐Akt (Ser473) (D9E) XP® Rabbit mAb	CST	4060
JNK antibody	Affinity	AF6318
Phospho‐JNK1/2/3 (Tyr185) Antibody	Affinity	AF3320
NF‐κB p65 (D14E12)	CST	8242S
Phospho‐NF‐κB p65 (Ser536) (93H1)	CST	3033S
p38 MAPK Antibody	AFFINITY	AF6456
Phospho‐p38 MAPK (Thr180/Tyr182) Antibody	AFFINITY	AF4001
GAPDH (D16H11) XP® Rabbit mAb	CST	5174S
HRP‐conjugated Affinipure Goat Anti‐Mouse IgM	Proteintech	SA00012‐6
**Bacterial strains**		
*Lactobacillus johnsonii* PYSLJ‐1	This paper	CGMCC NO.28168
**Chemicals, peptides, and recombinant proteins**		
Resiquimod	MCE	S28463
Murine IL‐4	Peprotech	214‐14
Human IL‐2	Peprotech	200‐02‐50
Metronidazole	MCE	HY‐B0318
Neomycin	MCE	HY‐150520
Ampicillin	MCE	HY‐B0522
Vancomycin	MCE	HY‐B0671
db‐cAMP	MCE	HY‐B0764
ZM241385	MCE	HY‐19532
H‐89 dihydrochloride	MCE	HY‐15979A
Rapamycin	MCE	HY‐10219
inosine	MCE	HY‐N0092
LW6	MCE	HY‐10219
PD98059	MCE	HY‐12028
KLH	Sigma‐Aldrich	9013‐72‐3
**Critical commercial assays**		
MojoSort™ Human Pan B Cell Isolation Kit	Biolegend	480082
MojoSort™ Mouse Pan B Cell Isolation Kit II	Biolegend	480088
ChamQ Universal SYBR qPCR Master Mix	Vazyme	Q711‐02
HiScript III RT SuperMix for qPCR (+gDNA wiper)	Vazyme	R323‐01
Mouse anti‐double stranded DNA antibody (IgG) ELISA Kit	CUSABIO	CSB‐E11194m
Mouse ANA (IgG) ELISA Kit	CUSABIO	CSB‐E12912m
Precision Plus Protein™ Dual Color Standards	BIO‐RAD	1610394
BASIC RPMI 1640 Medium	Gibco	C11875500CP
BCA Protein Assay Kit	Beyotime	P0011
**Oligonucleotides**		
spib Forward 5′‐AGGAGTCTTCTACGACCTGGA‐3′	PrimerBank	N/A
spib Reverse 5′‐GAAGGCTTCATAGGGAGCGAT‐3′	PrimerBank	N/A
Pax5 Forward 5′‐TCCCAGATGTAGTCCGCCAAA‐3′	PrimerBank	N/A
Pax5 Reverse 5′‐TCCTGTCTCATAATACCTGCCAA‐3′	PrimerBank	N/A
Il21 Forward 5′‐GGCTGCCTTACTCCTGCTG‐3′	PrimerBank	N/A
Il21 Reverse 5′‐TCATCTTGCCAGGTGAGACTG‐3′	PrimerBank	N/A
irf4 Forward 5′‐CCGACAGTGGTTGATCGACC‐3′	PrimerBank	N/A
irf4 Reverse 5′‐CCTCACGATTGTAGTCCTGCTT‐3′	PrimerBank	N/A
bach2 Forward 5′‐GAGGAAGGAGTTCCGAGCC‐3′	PrimerBank	N/A
bach2 Reverse 5′‐CAAGTCATCTTTCGTCTGTCCA‐3′	PrimerBank	N/A
cd38 Forward 5′‐TCTCTAGGAAAGCCCAGATCG‐3′	PrimerBank	N/A
cd38 Reverse 5′‐GTCCACACCAGGAGTGAGC‐3′	PrimerBank	N/A
bcl2 Forward 5′‐ACGTGGACCTCATGGAGTG‐3′	PrimerBank	N/A
bcl2 Reverse 5′‐TGTGTATAGCAATCCCAGGCA‐3′	PrimerBank	N/A
prdm1 Forward 5′‐AGTCCCAGGACAAGGCGAA‐3′	PrimerBank	N/A
prdm1 Reverse 5′‐GTGGTGGCGAGGTTCCTAA‐3′	PrimerBank	N/A
bcl6 Forward 5′‐CGCATGTGTGGCATCAACG‐3′	PrimerBank	N/A
bcl6 Reverse 5′‐TCCCAACATAGTCCATTTTTGGC‐3′	PrimerBank	N/A
S1pr1 Forward 5′‐ATGGTGTCCACTAGCATCCC‐3′	PrimerBank	N/A
S1pr1 Reverse 5′‐CGATGTTCAACTTGCCTGTGTAG‐3′	PrimerBank	N/A
ebi2 Forward 5′‐ATGGCTAACAATTTCACTACCCC‐3′	PrimerBank	N/A
ebi2 Reverse 5′‐ACCAGCCCAATGATGAAGACC‐3′	PrimerBank	N/A
ccr6 Forward 5′‐TGGGCCATGCTCCCTAGAA‐3′	PrimerBank	N/A
ccr6 Reverse 5′‐GGTGAGGACAAAGAGTATGTCTG‐3′	PrimerBank	N/A
ccr7 Forward 5′‐TGTACGAGTCGGTGTGCTTC‐3′	PrimerBank	N/A
ccr7 Reverse 5′‐GGTAGGTATCCGTCATGGTCTTG‐3′	PrimerBank	N/A
hif1a Forward 5′‐GTCCCAGCTACGAAGTTACAGC‐3′	PrimerBank	N/A
hif1a Reverse 5′‐CAGTGCAGGATACACAAGGTTT‐3′	PrimerBank	N/A
a1r Forward 5′‐TGTGCCCGGAAATGTACTGG‐3′	PrimerBank	N/A
a1r Reverse 5′‐TCTGTGGCCCAATGTTGATAAG‐3′	PrimerBank	N/A
a2ar Forward 5′‐GCCATCCCATTCGCCATCA‐3′	PrimerBank	N/A
a2ar Reverse 5′‐GCAATAGCCAAGAGGCTGAAGA‐3′	PrimerBank	N/A
a3r Forward 5′‐AAGGTGAAATCAGGTGTTGAGC‐3′	PrimerBank	N/A
a3r Reverse 5′‐AGGCAATAATGTTGCACGAGT‐3′	PrimerBank	N/A
ccl19 Forward 5′‐GGGGTGCTAATGATGCGGAA‐3′	PrimerBank	N/A
ccl19 Reverse 5′‐CCTTAGTGTGGTGAACACAACA‐3′	PrimerBank	N/A
CD11c Forward 5′‐CAAGAAGCACCGAACATGGTT‐3′	PrimerBank	N/A
CD11c Reverse 5′‐GTCTGAGCTAGAGTCACTGGT‐3′	PrimerBank	N/A
F4/80 Forward 5′‐TGACTCACCTTGTGGTCCTAA‐3′	PrimerBank	N/A
F4/80 Reverse 5′‐CTTCCCAGAATCCAGTCTTTCC‐3′	PrimerBank	N/A
β‐actin Forward 5′‐GTGACGTTGACATCCGTAAAGA‐3′	PrimerBank	N/A
β‐actin Reverse 5′‐GCCGGACTCATCGTACTCC‐3′	PrimerBank	N/A

To investigate whether the changes in purine metabolites were linked to the modulated gut microbiota, we conducted a correlation analysis between the metabolites involved in purine metabolism and the significantly differentiated microbial strains. Notably, the genus *Lactobacillus* strongly correlated with purine metabolites (Figure 5C). We also detected a notable increase in plasma inosine level after FMT treatment (Figure 5D). To further evaluate the relationship between *L. johnsonii* and inosine, GF mice were monocolonized with *L. johnsonii*. As a result, plasma inosine level was significantly increased (Figure 5E). This illustrated that *L. johnsonii* has the capacity to produce inosine. We also observed a notable decrease in serum inosine levels among SLE patients (Figure S10F, Supporting Information). While inosine levels displayed a negative correlation with the Systemic Lupus Erythematosus Disease Activity Index 2000 (SLEDAI‐2K) scores, this relationship did not reach statistical significance (Figure S10G, Supporting Information). These results indicated microbiota alteration contributed to purine metabolites enrichment, which was associated with autoimmunity alleviation.

We then asked whether administration of either *L. johnsonii* or its‐associated inosine is sufficient to protect mice from lupus pathogenesis. A 15‐week administration of *L. johnsonii* showed increased plasma inosine level in pristane‐induced mice (Figure 5F). Additionally, the administration of inosine significantly increased plasma inosine levels, which were comparable to those with FMT treatment (Figure 5D,F). Indeed, following 4‐week treatment with inosine, there was a significant reduction in anti‐dsDNA levels compared to the saline group (*p *< 0.01, Figure 5G). The *L. johnsonii*‐treatment achieved similar protection, albeit the reduction in dsDNA autoantibody levels did not reach statistical significance (*p* = 0.079, Figure 5G). Similar to our FMT results, immune profiling revealed an increasing trend in naïve T cells and a decreasing trend in memory T cells in CD4^+^ T cells (Figure 5H) and CD8^+^ T cells systemically (Figure 5H). Inosine and *L. johnsonii* treatment significantly increased the percentage of naïve B cells in draining lymph nodes (DLNs) (Figure 5I), and showed a decline tendency in memory B cells (Figure 5J). We also found a significant decrease in GCB percentage and cell number of GCB in *L. johnsonii* and inosine‐treated groups (Figure 5K). *L. johnsonii* and inosine improved the pathological score of the small intestines (Figure S11A,B, Supporting Information). In small intestines, inosine treatment significantly increased naïve B cells (*p *< 0.01), and decreased antibody‐secreting cells (ASCs) (*p *< 0.0001) and GCB (*p *< 0.0001), while there was a slight decrease in memory B cells but did not reach statistical significance (Figure S11C, Supporting Information). Furthermore, we observed a significant reduction in ASCs of MLNs by inosine treatment (Figure S11D, Supporting Information), along with a slight decline in Peyer's patches (PPs) (Figure S11D, Supporting Information). Additionally, *L. johnsonii* treatment significantly increases the frequency of Treg cells in small intestinal lymphocytes compared with the saline‐treated group (Figure S11E, Supporting Information). Moreover, we observed that gut barrier integrity was improved by *L. johnsonii* treatment (Figure S11F, Supporting Information).

As a consequence, both inosine and *L. johnsonii* lowed the urine protein levels in pristane‐induced lupus mice compared to the saline group (Figure S12A–C, Supporting Information). The ratio of urine protein to creatinine decreased after 13 weeks of inosine treatment, with a slight decrease being also noted in the *L. johnsonii*‐treated group after 13 weeks (Figure S12B, Supporting Information). Histology analysis revealed that *L. johnsonii* and inosine intervention reduced immune cell infiltration, ameliorated glomerulonephritis, and decreased interstitial fibrosis (Figure S12D, Supporting Information). Lower levels of IgG, IgG2a, and C3 deposition were detected in kidneys compared to those in saline‐treated mice (Figure S12E, Supporting Information). *L. johnsonii* treatment significantly reduced *Cd38*, *Ccr7*, and *S1pr1* level in kidney, while inosine intervention exhibited *Ccr7* and *Ebi2* reduction in mouse kidney (Figure S12F, Supporting Information). Both interventions also showed a decreasing trend in *Cd19* expression (Figure S12F, Supporting Information). Collectively, our data showed that *L. johnsonii* and inosine treatment ameliorated LN in pristane‐induced mice.

We then confirmed the protective effects of *L. johnsonii* and inosine in MRL/lpr mice (Figure S13A, Supporting Information). Consistently, both *L. johnsonii* and inosine reduced anti‐dsDNA IgG levels (Figure S13B, Supporting Information), with a minor reduction in the urine protein levels compared with the saline group (Figure S13C, Supporting Information). Flow cytometry analysis confirmed a significant increase in the percentage of naïve B cells by *L. johnsonii* and inosine treatment (Figure S13D, Supporting Information). *L. johnsonii* markedly decreased the percentage of ASCs, while inosine markedly reduced the plasmablasts in MRL/lpr mice (Figure S13D, Supporting Information). These findings collectively suggested that both inosine and *L. johnsonii* hold potent protective effects on suppressing autoimmune responses through suppressing B cell activation and differentiation.

Collectively, fecal metabolite profiles were influenced by changes in the gut microbiome, and purine metabolism emerged as the most prominently enriched pathway following FMT treatment. Our findings indicate that inosine, a purine metabolite, showed a significant increase of 3.85‐fold in feces (Figure S10E, Supporting Information) and 1.82‐fold in plasma after FMT treatment (Figure 5C). Additionally, inosine levels were also elevated in plasma with *L. johnsonii* monocolonization. These results suggest that *L. johnsonii* may exert its effects on controlling autoimmunity in lupus mice through modulating gut microbiota homeostasis and involving in the metabolite inosine.

### 2.7 Inosine Inhibited KLH‐Specific Immune Response

To further investigate the impact of inosine on the antigen‐specific B‐cell response, we treated the keyhole limpet hemocyanin (KLH)‐immunized C57BL/6j mice with either saline or inosine (Figure S14A, Supporting Information). Immune cells and serum were collected on day 21 after immunization (Figure S14A, Supporting Information). Compared with the saline‐treated group, the frequency of B220^+^ and CD19^+^ cells in the spleen showed a slight decrease with inosine treatment (Figure S14B, Supporting Information). Additionally, an increase in CD4^+^ naïve T cells was observed in the spleen of mice treated with inosine. Inosine significantly increased the population of CD8^+^ naïve T cells while decreasing CD8^+^ memory T cells in the spleen (Figure S14C, Supporting Information). Furthermore, the inosine‐treated group exhibited a higher frequency of naïve B cells in the DLNs and a lower frequency of memory B cells in the spleen and the DLNs (Figure S14D, Supporting Information). Additionally, inosine treatment significantly decreased the levels of KLH‐specific IgG1 in the plasma on day 14 and day 21. Still, the other types of antibodies, including IgG2a, IgG2b, IgG3, and IgM, were unaltered at different time points (Figure S14E, Supporting Information).

### 2.8 Inosine Restricted B Cell Differentiation via ERK‐HIF‐1α Signaling Pathway

**Figure 6:**
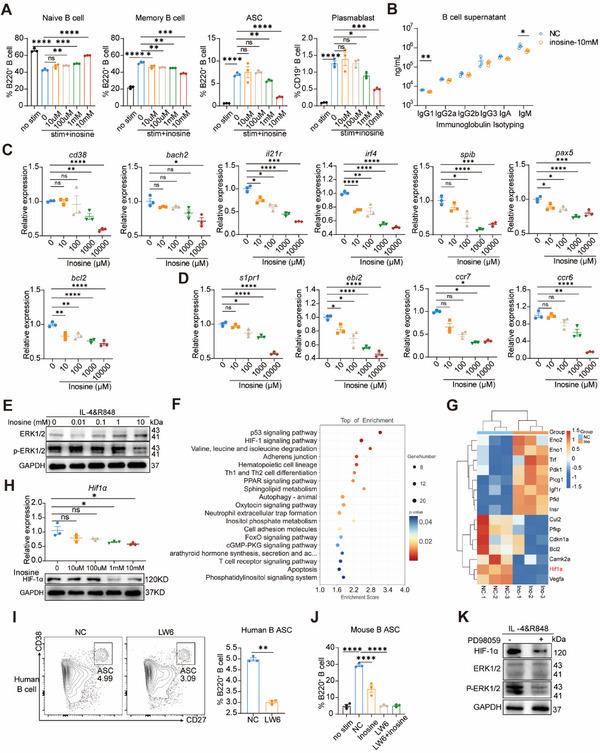
Inosine restricted B cell differentiation in vitro. A) Isolated mouse splenic CD19^+^ B cells were triggered by IL‐4 and R848 incubated with different concentrations of inosine (0, 10 µm, 100 µm, 1 mm, 10 mm) for 48 h. Statistical analysis of frequencies of naive B cells, memory B cells, ASC, and plasmablast with different concentrations of inosine. B) Ig isotyping in mouse B cell supernatant treated with 10 mm inosine. C) The mRNA level of genes related to B cell differentiation, including *spib, pax5, il21r, bach2, irf4, cd38* and *bcl2, prdm1*, and *bcl6*. D) The mRNA level of genes related to B cell migration, including *s1pr1, ebi2, ccr7*, and *ccr6*. E) Western blot detected the ERK pathway after increased inosine treatment in mouse splenic B cells after 48 h incubation. F) GO‐KEGG enrichment analysis of the differentially expressed genes in RNA‐seq. G) Differential expressed genes in HIF signaling pathway. H) The mRNA level of *hif1α* and protein level of HIF‐1α in mouse spleen B cells with different concentrations of inosine (0, 10 µm, 100 µm, 1 mm, 10 mm). I) Representative figures and statistical analysis of frequencies of ASC with or without LW6 (10 µm) in human peripheral blood B cells culture for 48 h. J) Frequencies of ASC with or without LW6 (10 µm) or inosine in mouse splenic B cells cultured for 48h. K) Western blot for HIF‐1α protein with or without PD98059 treatment in mouse splenic B cells after 48 h incubation (20 µm). Data were obtained from three biologically replicated experiments. The results are expressed as mean ± SEM. Statistical comparison was based on one‐way ANOVA. *p < 0.05 was considered statistically significant; **p < 0.01; ***p < 0.001; ****p < 0.0001; ns = not significant.

We next investigated the mechanisms underlying the impact of inosine on B cell immunity. Mouse splenic B cells were isolated and stimulated with IL‐4 and TLR7/8 agonist R848 in the presence of varying concentrations of inosine.^[^ ^32^ ^]^ Inosine dose‐dependently decreased the frequency of memory B cells, with a consequent increase of naïve B cells (**Figure** **6**A). Inosine significantly reduced the percentage of ASCs at the dose of 1 mm (*p* < 0.01) and 10 mM (*p *< 0.0001) (Figure 6A). Similarly, the percentage of plasmablasts showed a significant decrease by inosine treatment at the concentrations of 1 mm (*p* < 0.05) and 10 mm (*p *< 0.001) (Figure 6A). Consistently, the concentration of IgG1 (*p *< 0.01) and IgM (*p *< 0.05) was significantly decreased by the 10 mm inosine treatment, and IgA also exhibited a noticeable decrease, although it did not reach statistical significance (Figure 6B). The mRNA levels of *cd38, bach2*, and *bcl2*, which are associated with memory B cell generation, were significantly decreased in response to inosine treatment (Figure 6C). The transcription factor SPI‐B is crucial for GC initiation, whereas PAX5 and IL‐21R are responsible for maintaining GCB cell identity or promoting differentiation into memory B cells.^[^ ^33^, ^34^ ^]^ We observed a gradual decrease in the expression levels of *il21r, spib*, and *pax5* in B cells when treated with the increased concentration of inosine (Figure 6C). Additionally, interferon regulatory factor 4 (IRF4), which plays a role in promoting the differentiation of cells into plasma cells, exhibited a significant decrease by inosine treatment in a dose‐dependent manner (Figure 6C). Moreover, inosine reduced the mRNA levels of *s1pr1, ebi2, ccr7* and *ccr6*, which are involved in B cell migration (Figure 6D). Therefore, inosine inhibits the expression of a set of critical genes for B cell immunity. These results demonstrated that the *L. johnsonii*‐associated metabolite inosine inhibits B cell activation, differentiation, and migration in vitro.

We then explored the mechanism through which inosine restricting B cell differentiation and migration. It has been shown that inosine binds to the adenosine 2A receptor (A_2A_R), a receptor known to exert inhibitory effects on Th1 differentiation.^[^ ^12^, ^35^, ^36^ ^]^ The mRNA levels of *A_2A_R* and *A_3_R* gradually decreased with increasing inosine concentrations, while there was no significant inhibitory effect on *A_1_R* and *A_2B_R* mRNA levels (Figure S15A, Supporting Information). However, pharmacological inhibition of A_2A_R signaling using ZM241385 did not abrogate the effect of inosine on B cell differentiation (Figure S15B, Supporting Information), nor by the cell‐permeable cyclic AMP (db‐cAMP), a signaling molecule downstream of A_2A_R (Figure S15B, Supporting Information). Additionally, the inhibition of protein kinase A (PKA), a downstream effector molecule of cAMP, enhanced the inhibitory effect of inosine on B cell differentiation (Figure S15B, Supporting Information). Since the mammalian target of rapamycin (mTOR) pathway is known to modulate B cell differentiation,^[^ ^37^ ^]^ we also investigated whether inosine affected B cells through mTOR. Similarly, adding rapamycin, a known inhibitor of mTORC1, also enhanced the restriction of inosine on B cell differentiation (Figure S15B, Supporting Information). Collectively, our data indicated that neither A_2A_R‐cAMP‐PKA signaling nor the mTOR pathway is responsible for inosine to restrict B cell differentiation and migration.

We then explored alternative pathways through which inosine might influence B cells. Western blot analysis showed that inosine decreased the levels of phosphorylated MAPK member ERK1/2 (Figure 6E). Conversely, it increased the phosphorylation level of p38 MAPK (Figure S16A, Supporting Information). However, the inhibitor of p38 MAPK, SB 203580, promoted the impact of inosine's function on B cell (Figure S16B, Supporting Information). Moreover, the phosphorylation levels of protein kinase B (AKT1), the c‐Jun N‐terminal kinase (JNK), and nuclear factor‐kB p65 showed no obvious change (Figure S16A, Supporting Information). We found an enrichment of hypoxia‐inducible factor (HIF) signaling pathway (Figure 6F). We further identified differentially expressed genes in HIF signaling pathway, and found *hif1α* significantly decreased with inosine treatment (Figure 6G).

It has been reported that HIF‐1α is essential for B cell immunity.^[^ ^38^, ^39^, ^40^, ^41^ ^]^ We observed a gradual decrease in both mRNA and protein levels of HIF‐1α in B cells with increasing concentration of inosine treatment (Figure 6H). Additionally, inosine also reduced HIF‐1α expression in human B cells (Figure S16C, Supporting Information). Furthermore, the inhibitor of HIF‐1α, LW6, significantly reduced the percentage of ASCs in mouse B cells and human peripheral blood B cells (Figure 6I,J), while inhibiting HIF‐1α largely abolished the function of inosine on B cells (Figure 6J). These data revealed that the effects of inosine on B cells were involved in modulating HIF‐1α. Treatment with the ERK1/2 inhibitor PD98059 resulted in a reduction of HIF‐1α expression in B cells (Figure 6K). These results imply that the selective inhibition of ERK‐ HIF‐1α pathways is involved in inosine‐mediated suppression of B cell immunity.

Therefore, we have demonstrated the inhibitory effects of microbial‐derived inosine on B cell function by reducing the expression of multiple genes related to B cell differentiation and migration. Mechanistically, inosine inhibits the ERK1/2 signaling pathway, suppressing downstream HIF‐1α, possibly inhibiting B cell activity.

## 3 Discussion

In this study, we demonstrated that FMT from healthy mice to lupus mice led to significant improvements in lupus‐like symptoms, with a notable reduction of anti‐dsDNA antibody secretion, a pivotal hallmark of lupus progression. FMT positively affected autoimmunity by diminishing CSR and elevating IGH naïve isotypes via BCR sequencing. Furthermore, improving gut microbiota dysbiosis alleviated kidney damage by reducing B cells and macrophage infiltration. These data support the notion that altering the microbiota or employing precise bacterial interventions involving well‐defined microbial communities may provide a promising therapy for systemic autoimmunity. Indeed, the safety and efficacy of FMT for the treatment of SLE patients have been investigated in a single‐arm pilot clinical trial, leading to a notable decrease in SLEDAI‐2K scores and reduced the level of serum anti‐dsDNA antibody compared to baseline.^[^ ^42^ ^]^ This clinical trial in active SLE patients provides supportive evidence that altered gut microbiome and metabolite profiles can be a viable, safe, and potentially effective therapy in SLE patients.^[^ ^42^ ^]^ Despite the finding that the gut microbiome regulates lupus‐associated immune responses, the precise molecular mechanisms by which microbes and microbiota‐derived metabolite(s) affect lupus therapy remain elusive. Furthermore, we identified an *L. johnsonii* strain after FMT as a key commensal intestinal bacterial species, exhibiting a robust correlation with purine metabolites. Increasing *Lactobacillus* in the gut alleviated lupus‐like phenotype by regulating the ratio of regulatory versus pathogenic T cells.^[^ ^43^ ^]^ A synergy of five Lactobacillus strains (*L. oris, L. rhamnosus, L. reuteri, L. johnsonii, and L. gasseri*) has the potential to ameliorate LN by reducing the deposition of IgG2a in the kidneys.^[^ ^43^, ^44^ ^]^ Although isolated from mice, *L. johnsonii* is a probiotic suitable for humans, suggesting its potential for translation.^[^ ^45^, ^46^, ^47^ ^]^ Moreover, we dug out the enriched purine metabolites following FMT, strongly correlated with *L. johnsonii* strains. Inosine, the intermediate product in purine biosynthesis and a secondary metabolite of purine degradation, exhibited a 3.85‐fold increase post FMT. The supplementation of *L. johnsonii* and inosine ameliorated lupus‐like symptoms in the mouse model.

Furthermore, we analyzed a published human fecal metabolic dataset in SLE patients and found that purine metabolite adenosine was significantly decreased (up to 2.7‐fold; Figure S17A, Supporting Information) in SLE patients compared to that in healthy control. Additionally, this decrease showed a modest negative correlation with SLEDAI‐2K scores (Figure S17B,^[^ ^48^ ^]^ Supporting Information). Although inosine displayed only a minor reduction in SLE patients in this cohort (Figure S17C, Supporting Information), we observed a distinct negative correlation with SLEDAI‐2K scores (Figure S17D, Supporting Information). Inosine has been reported to inhibit in vitro differentiation of Th1 and Th2 cells, depending on adenosine A2a receptors.^[^ ^12^, ^49^ ^]^ Furthermore, in co‐stimulation, inosine can enhance the development of DC‐dependent Th1 cells.^[^ ^13^ ^]^ Despite its intricate role in T cell differentiation, microbiota‐derived inosine plays a pivotal role in the modulation of adaptive immunity. We identified inosine, a key increased bacterial‐derived metabolite that increased post‐FMT and acted through ERK signaling to restrict B cell differentiation and reduce IgG antibody secretion by diminishing HIF‐1α expression. This study elucidated the possible bacteria and metabolites that play crucial roles in reducing inflammation and regulating the immune response after FMT, which provides a novel treatment strategy for SLE based on specific gut bacteria and microbiota‐related metabolites (Figure S18, Supporting Information). Further investigation of the effects of other purine metabolites, including purine, adenosine, xanthine, and hypoxanthine, is warranted.

### 3.1 Conclusion

In this study, we showed that FMT exerted a beneficial effect on autoimmunity by diminishing CSR and elevating IGH naïve isotypes via BCR sequencing. B‐cell infiltration in mouse kidneys was markedly reduced when reversing gut microbial dysbiosis, which contributed to alleviating LN. High‐throughput rDNA seq revealed significant alterations in microbiome structures following FMT, characterized by an increase in Lactobacillus taxa, with L. johnsonii emerging as the dominant species. Importantly, L. johnsonii exhibited a robust correlation with purine metabolites, particularly inosine, as the most pronounced increase post‐FMT. Administration of inosine effectively alleviated lupus‐like symptoms in mouse models by suppressing B cell differentiation, diminishing autoantibody secretion, and reducing renal B cell infiltration. Mechanically, inosine possibly inhibited B cell differentiation via ERK‐HIF‐1α signalling pathways. Our study highlights the discovery of a novel microbial metabolite that inhibits B cell autoimmunity, paving the way for innovative microbiome‐based therapeutic approaches.

## 4 Experimental Section

### Human Subjects

All human subjects were approved by the ethics committee of the Institute of Dermatology, Chinese Academy of Medical Sciences (2021.054). Serum samples were collected from SLE patients (*n* = 28) and age‐matched healthy controls (*n* = 14). The patients were with a diagnosis of SLE according to European League Against Rheumatism (EULAR)/American College of Rheumatology (ACR) 2019 classification criteria for SLE.^[^ ^50^ ^]^

### Animals

Eight‐week‐old female MRL/lpr, MRL/mpj, and C57Bl/6j mice were utilized in this study unless otherwise indicated in the figures. The mice were kept in a specific pathogen‐free (SPF) environment, with unrestricted access to water and a conventional laboratory diet unless otherwise specified. They were kept on wood chips and were housed in a room with a temperature‐controlled air conditioner and a light‐dark cycle of 12 h. Prior to the start of the study, all mice were in good health, had not undergone any previous procedures, and were drug‐naive. The mice were cohabitated for a duration of 2 weeks inside experimental enclosures to establish a balance in the microbiomes originating from various cages and parental sources. Female C57Bl/6j mice were intraperitoneally injected with 500 µL pristane (Sigma, P9622). The animal ethical and care guidelines of the Institute of Dermatology, Chinese Academy of Medical Sciences (no. 2022‐DW‐017) were adhered to and local government authorities’ approval was obtained.

### Method Details—Antibiotic Treatment

A cocktail of antibiotics containing metronidazole (0.5 g L⁻^1^; MCE), neomycin (0.5 g L⁻^1^; MCE), ampicillin (0.5 g L⁻^1^; MCE), and vancomycin (0.5 g L⁻^1^; MCE) was used in the drinking water for 12 weeks or a targeted regimen for 2 weeks and then replaced with regular water for the duration of the experiment. Sweetener (Equal, 2 g L⁻^1^) was added to both the antibiotics and control water due to the metallic taste of metronidazole.

### Fecal Microbiota Transplantation

Mice were cohoused for 2 weeks and then given antibiotics for 2 weeks to induce a gastrointestinal purge. The mice underwent fecal microbiota transplantation (FMT) whereas the control group was orally administered with saline. Detailly, fresh pellets were collected and sampled from MRL/mpj mice throughout the age ranging from 8 to 30 weeks. The collected feces were then pooled and suspended in sterile saline using a vortex. The suspension of feces was filtered through a 100‐µm sieve. After collecting the gut microbiota by centrifugation, the pellets were suspended and administered at a concentration of 0.1 mg mL⁻^1^ to the recipient mice. The mice were orally gavaged with 300 µL⁻^1^/times, 3 times/week.

### Bacterial Culture and Stock

*L. johnsonii* was isolated from feces in MRL/mpj mice. This strain was named as *L. johnsonii* PYSLJ‐1 and sent to China Microbiological Culture Preservation and Management Committee for preservation (No. CGMCC NO.28168). The fresh feces were placed and homogenized with steel homogenizer in 1 mL Gifu Anaerobic Medium (Haibo, Fluid). Diluted (10^−4^–10^−5^) samples were plated on Gifu Anaerobic Media agar plates (Haibo) resulting in distinct colonies (≈200 colonies per plate) after 72 h of incubation at 37 °C in an anaerobic chamber. Single colonies were harvested and re‐streaked on GAM agar plates for ≈48 h at 37 °C in an anaerobic chamber. The bacterial species was assessed by Matrix‐Assisted laser Desorption/lionization Time of Flight Mass Spectrometry (MALDI‐TOFMS). Stocks were generated in glycerol (final concentration was 15%) and stored in anaerobic glass vials in a standard − 80 °C freezer.

### *L. Johnsonii* and Inosine Oral Gavage Experiments

Pristane‐induced C57BL/6 mice and MRL/lpr mice were colonized by oral gavage with 2 × 10^9^ CFU of *L. johnsonii*, or 50 mg kg^−1^ inosine (MCE, HY‐N0092), 300 µL⁻^1^/times, 3 times/week. Animals were sacrificed, and spleen, DLNs, MLNs, PPs, and kidneys were collected.

### Monocolonization with *L. Johnsonii* in Germ‐Free Mice

GF C57BL/6 mice (6‐8 weeks) were bred and maintained in special plastic isolators (GemPharmatech, Nanjing). All mice were housed under a strict 12‐h light cycle (lights on at 08:00). Animals were supplied with a 50‐kGy irradiated sterile pelleted normal chow diet (Xietong Shengwu, Nanjing) and autoclaved tap water ab libitum. Bedding was replaced in all experiments every 7 days. All GF mice were routinely screened for bacteria, viral, and fungus contamination.

### Gut Permeability Detection Assay with FITC‐Dextran In Vivo

Mice were weighed and fasted for 4 h prior to oral FITC‐dextran (4 KDa; Ruixibio) administration. Through oral gavage, a total concentration of 250 mg kg^−1^ body weight was administered. After 3 h of the administration, blood samples were collected through the orbital vein and 50 µL plasma was placed in a fluorescence plate reader to determine the concentration by fluorescence excitation at 495/520 nm reference.

### Assessment of Bacterial Translocation

Liver, kidney, and MLN were aseptically collected and used for isolation of anaerobic or aerobic bacteria. Tissue pieces were homogenized with steel homogenizer in 1 mL Gifu Anaerobic Medium (Haibo, Fluid) under sterile conditions. PBS was used as tissue surrogate and went through the same workflow to evaluate the environmental contaminants. 100 uL sample homogenate was plated on Gifu Anaerobic Medium agar and incubated for 72 h at 37 °C with 5% CO_2_ aerobically and anaerobically.

### Proteinuria and Urine Creatinine

Urine from pristane‐induced mice and MRL/lpr mice was examined semiquantitatively and quantitatively for proteinuria using the test strips (Urit) and a Coomassie Brilliant Blue reagent (CBB; Njjcbio), respectively. Creatinine in urine was determined using a creatinine assay reagent (sarcosine oxidase; Njjcbio).

### Cytometric Bead Assay

Plasma samples from pristane‐induced mice and KLH‐immunized mice and supernatants from cultured splenic B cells were collected and subjected to analysis using a LEGENDplex™ Mouse Immunoglobulin Isotyping Panel (6‐plex) (BioLegend, 740 492), following the manufacturer's protocols. The different isotypes of mouse immunoglobulins (IgA, IgM, IgG1, IgG2a, IgG2b, and IgG3) levels were determined using LEGENDplex software after flow cytometry analysis on a BD FACSVerse™ Flow Cytometer instrument.

### Histopathology

Tissues were dissected at necropsy and fixed in 4% paraformaldehyde (PFA), and histological sections were stained with hematoxylin and eosin (H&E). The severity of glomerulonephritis and interstitial nephritis was scored in a blinded fashion on a scale of 0–4 based on the intensity and extent of histopathological change as previously described.^[^ ^51^ ^]^

### Enzyme‐Linked Immunosorbent Assays (ELISA)

Plasma antibodies specific for dsDNA (CUSABIO) and ANA (CUSABIO) were determined using standardized ELISAs followed the instructions. Serum was diluted 1:20 for anti‐dsDNA antibody and 1:200 for ANA. ELISA kits were used for serum inosine in healthy control and SLE patients (mlbio), plasma inosine in mice (senbeijia). Serum was diluted 1:5 and incubated for 1 h at 37 °C. 2 m sulfuric acid solution was used to stop the reaction. Concentrations of all antibodies were determined by reading the absorbance at 450/570 nm reference.

### Immunofluorescence Studies

Immunofluorescence (IF) was conducted on kidney sections embedded in OCT compound (Sakura) to detect IgG depositions, utilizing Goat Anti‐Rabbit IgG (FITC) (Proteintech, SA00003‐2, 1:200) and Goat Anti‐Mouse IgG2a heavy chain (FITC) (Abcam, ab97244, 1:500). C3 deposits were detected using C3/C3b/C3c Polyclonal antibody (Proteintech, 21337‐1‐AP, 1:500) and Goat Anti‐Rabbit IgG (FITC) (Proteintech, SA00003‐2, 1:200) by IF.

### Immune Repertoire

Spleens were collected from pristane‐induced mice, and RNA was extracted using TRIzol Reagent (Meridian Life Science, US). RNA quality and quantity were detected using a NanoDrop ND‐2000 spectrophotometer. All RNA samples met the requirements of IR sequencing on concentration and purity, and the volume of each sample was consistent (the total RNA content needed to be 500 ng). Amplification and sequencing were completed using the Illumina Nova‐Seq platform. Next, the raw sequences obtained were collapsed by 10‐bp UMI tags into the consensus FASTA format using MiGEC version 1.2.9 (https://migec.readthedocs.io/en/latest/) and were analyzed using the iRmap program.^[^ ^52^, ^53^ ^]^ Details of this IR methodology are described by Niu et al.^[^ ^54^ ^]^ For immune‐related genes sequencing, paired‐end fastqs were demultiplexed by 6 bp barcode using MiGEC version 1.2.9 and then stitched into a single read using pandaseq version 2.11. The resulting fastq was aligned to a set of reference sequences based on a custom panel of immune‐related genes using bowtie2 with the very‐sensitive‐local parameter. Gene summary data were created by counting the number of aligned reads for each gene.

### Single‐Cell RNA Sequencing Data Analysis—Data Processing

Cellranger (V.3.0) was applied for data quantification, primary quality control, and read alignment. The R package Seurat (V4.1.3) was used to merge Seurat object of each sample. To realize further quality control, cells were filtered out with > 20 000 transcripts, < 300 genes, > 4 000 genes, or more than 20% of mitochondrial expression. Then, high‐quality cells were collected for NomalizeData to standardize the gene expression. The FindVariableFeature function was performed to calculate highly variable genes (HVGs), and the Harmony package to remove the batch effect. The ScaleData function was used to scale and center the counts in the dataset. Dimensionality reduction and cell clustering were conducted by FindNeighbors, FindClusters, and RunUMAP functions with the first 15 principal components (PC) and 0.5 resolution. The differentially expressed genes (DEGs) of cell clusters were calculated using the FindAllMarkers function. The classic cell type marker genes were used to annotate each cell cluster.

### Single‐Cell RNA Sequencing Data Analysis—GO Analysis

At first, DEGs of each cell cluster or group using FindAllMarkers were obtained. Then, the R package clusterProfiler was utilized to perform the GO enrichment analysis for the biological process. The *p*‐value of the meaningful enrichment pathway was set to be less than 0.01, and the *q*‐value to be less than 0.05.

### Single‐Cell RNA Sequencing Data Analysis—Data and Code Availability

The scRNA‐seq data exhibited in this paper can be accessed in the GEO database under accession number. All relevant data are publicly available as of the date of publication. Other information or data reported in this paper would be available upon reasonable request.

### Lymphocytes Isolation from Small Intestines Lamina Propria

Mice were euthanized and small intestines were collected and placed in ice‐cold cHBSS (HBSS containing 2% fetal calf serum). After getting rid of the fat, the lumen of the intestine was flushed with ice‐cold cHBSS. PPs were excised and collected. Intestines were cut longitudinally in a 10‐cm in 50‐mL Falcon tube containing cold PBS and mixed on a vortex for 10 s. Tissue was washed twice with 20 mL of HBSS.

Next, the small intestine was processed using the following digestion steps: small intestine was transferred to a 50‐mL Falcon tube with cHBSS (containing 10% FCS, 5 mm EDTA). The tubes were sealed and incubated at 37 °C for 30 min while being rotated every 10 min. After incubation, tubes were vortex and supernatants were collected and transferred through a 40‐µm strainer. After a washing step, the tissue was placed in a 15‐cm Falcon tube at room temperature and 5 mL of cHBSS containing collagenase D (1 mg mL⁻^1^, Roche) and DNAse I (0.1 mg mL⁻^1^, Roche) was added. Next, small intestine was minced into small pieces and incubated at 37 °C for 30 min while being rotated every 10 min. Samples were filtered through a 100‐µm strainer and washed in 10 mL of cHBSS. Then, cells were suspended in 5 mL of 40% percoll (Cytiva) and transferred to 3 mL 80% percoll. The mixture was centrifuged at a speed of 800 x g for 20 min at 4 °C with speed up 5 and slowdown 0. Lymphocytes were collected from the mesosphere. Finally, the cells were resuspended in 1 mL of media.

### Flow Cytometry

Cells isolated from spleen, DLN, MLN, small intestinal lymphocytes, or PP were used for surface‐staining or intracellular cytokines detection. Approximately 1 × 10^6^ cells were treated with mouse Fc‐R block (BD Pharmingen, 553 141) for 15 min at room temperature, followed by surface staining using specific marker antibodies for 45 min at 4 °C in the dark. Intracellular staining was implemented by fixing and permeabilizing the cells with a Transcription Factor Buffer Set (BD Pharmingen, 562 574) and staining with fluorescent antibodies for 1.5 h at 4 °C. To assess cytokine production by lymphocytes in the spleens, 2 × 10^6^ cells/well were stimulated with Leukocyte Activation Cocktail (BD GolgiPlug™ antibody, BD 550 583) in 6‐well plates for 6 h at 37 °C. Flow cytometry result was evaluated through FlowJo software version 10.8.1. Flow cytometry was performed using a Cytek NL‐CLC V16B14R8 instrument.

### 16S rDNA High‐Throughput Sequencing

Fecal samples were snap frozen and stored at − 80 °C after collection. Bacterial DNA was isolated from the caecal contents using a DNeasy PowerSoil kit (Qiagen, Hilden, Germany) following the manufacturer's instructions. PCR amplification of the V3‐V4 hypervariable regions of the bacterial 16S rRNA gene was carried out in a 25 µL reaction using universal primer pairs (343F: 5′‐TACGGRAGGCAGCAG‐3′; 798R: 5′‐AGGGTATCTAATCCT‐3′). Sequencing was performed on an Illumina NovaSeq6000 with two paired‐end read cycles of 250 bases each (Illumina Inc., San Diego, CA; OE Biotech Company; Shanghai, China). Paired‐end reads were filtering low quality sequences, denoised, merged and detect and cut off the chimera reads using DADA2^[^ ^55^ ^]^ with the default parameters of QIIME2.^[^ ^56^ ^]^ The microbial diversity in Caecal content samples was estimated using the alpha diversity that include Chao1 index and Shannon index. The Unifrac distance matrix performed by QIIME software was used for unweighted Unifrac Principal coordinates analysis (PCoA) and phylogenetic tree construction. The 16S rRNA gene amplicon sequencing and analysis were conducted by OE Biotech Co., Ltd. (Shanghai, China).

### Metagenomic Sequencing

Fecal samples from pristane‐induced mice were snap frozen and stored at − 80 °C after collection. The genomic DNA was randomly sheared into short fragments. The obtained fragments were end repaired, A‐tailed, and further ligated with Illumina adapter. The fragments with adapters were PCR amplified, size selected, and purified. The library was checked with Qubit and real‐time PCR for quantification and bioanalyzer for size distribution detection. Quantified libraries will be pooled and sequenced on Illumina platforms, according to effective library concentration and data amount required.

### Untargeted Metabolomics

Fecal samples from pristane‐induced mice were snap frozen and stored at − 80 °C after collection. Samples were individually ground with liquid nitrogen, and the homogenate was resuspended with prechilled 80% methanol by well vortex. The samples were incubated on ice for 5 min and then were centrifuged at 15 000 g, 4 °C for 20 min. Some of supernatant was diluted to final concentration containing 53% methanol by LC‐MS grade water. The samples were subsequently transferred to a fresh Eppendorf tube and then were centrifuged at 15 000 g, 4 °C for 20 min. Finally, the supernatant was injected into the LC‐MS/MS system analysis. UHPLC‐MS/MS analyses were performed using a Vanquish UHPLC system (Thermo Fisher, Germany) coupled with an Orbitrap Q ExactiveTMHF‐X mass spectrometer (Thermo Fisher, Germany) in Novogene Co., Ltd. (Beijing, China). Samples were injected onto a Hypesil Gold column (100 × 2.1 mm, 1.9 µm) using a 12‐min linear gradient at a flow rate of 0.2 mL min^−1^. The eluents for the positive polarity mode were eluent A (0.1% FA in Water) and eluent B (Methanol). The eluents for the negative polarity mode were eluent A (5 mm ammonium acetate, pH 9.0) and eluent B (Methanol). The solvent gradient was set as follows: 2% B, 1.5 min; 2–85% B, 3 min; 85–100% B, 10 min;100–2% B, 10 min;2% B, 12 min. Q ExactiveTM HF‐X mass spectrometer was operated in positive/negative polarity mode with spray voltage of 3.5 kV, capillary temperature of 320 °C, sheath gas flow rate of 35 psi, aux gas flow rate of 10 L min^−1^, S‐lens RF level of 60, and Aux gas heater temperature of 350 °C.

The raw data files generated by UHPLC‐MS/MS were processed using the Compound Discoverer 3.1 (CD3.1, Thermo Fisher) to perform peak alignment, peak picking, and quantitation for each metabolite. The main parameters were set as follows: retention time tolerance, 0.2 minutes; actual mass tolerance, 5 ppm; signal intensity tolerance, 30%; signal/noise ratio, 3; and minimum intensity, et al. After that, peak intensities were normalized to the total spectral intensity. The normalized data was used to predict the molecular formula based on additive ions, molecular ion peaks, and fragment ions. And then peaks were matched with the mzCloud (https://www.mzcloud.org/), mzVault, and MassList database to obtain the accurate qualitative and relative quantitative results. Statistical analyses were performed using the statistical software R (R version R3.4.3), Python (Python 2.7.6 version), and CentOS (CentOS release 6.6). When data were not normally distributed, normal transformations were attempted using of area normalization method.

These metabolites were annotated using the KEGG database (https://www.genome.jp/kegg/pathway.html), HMDB database (https://hmdb.ca/metabolites), and LIPIDMaps database (http://www.lipidmaps.org/). Principal components analysis (PCA) and Partial least squares discriminant analysis (PLS‐DA) were performed at metaX (metaX: a flexible and comprehensive software for processing metabolomics data) (a flexible and comprehensive software for processing metabolomics data). We applied univariate analysis (t‐test) to calculate the statistical significance (*p*‐value). The metabolites with VIP > 1 and *p*‐value < 0.05 and fold change ≥ 2 or FC ≤ 0.5 were considered to be differential metabolites. Volcano plots were used to filter metabolites of interest which based on log2 (FoldChange) and ‐log10 (*p*‐value) of metabolites by ggplot2 in R language.

For clustering heat maps, the data were normalized using z‐scores of the intensity areas of differential metabolites and were ploted by Pheatmap package in R language. The correlation between differential metabolites was analyzed by cor () in R language (method = pearson). Statistically significant of correlation between differential metabolites was calculated by cor.mtest() in R language. *p*‐value < 0.05 was considered as statistically significant, and correlation plots were plotted by corrplot package in R language. The functions of these metabolites and metabolic pathways were studied using the KEGG database. The metabolic pathways enrichment of differential metabolites was performed. When ratio was satisfied by x/n > y/N, metabolic pathway was considered as enrichment; when *p*‐value of metabolic pathway is < 0.05, metabolic pathway was considered as statistically significant enrichment.

### KLH Immunization

8‐week C57BL/6 mice except the Blank group were immunized with 200 µL KLH (0.5 mg mL⁻^1^) (Sigma) emulsified in CFA via subcutaneous injection on days 0 and 7. After immunization, the mice were sacrificed, and the lymph nodes, spleen tissues, and plasma samples were collected. The lymph nodes and spleen tissues were analyzed by flow cytometry. The plasma KLH‐specific total IgG1, IgG2a, IgG2b, IgG3, and IgM antibody levels were measured by ELISA.

### Culture of Splenic B cell

To obtain Mouse Pan B cells, splenocytes from C57BL/6j mice were isolated and purified through the MojoSort™ Mouse Pan B Cell Isolation Kit II (BioLegend, 480 088). The purified B cells were subsequently cultured in RPMI 1640 (Gibco, 21 870 076) with the addition of IL‐4 (PeproTech, 214‐14‐20, 30 ng mL⁻^1^) and R848 (MCE, S28463, 100 ng mL⁻^1^). In some conditions, cells were additionally cultured with various combinations of 100 µm db‐cAMP (MCE, HY‐B0764), 5 µm ZM241385 (MCE, HY‐19532), 30 µm H‐89 dihydrochloride (MCE, HY‐15979A) or 1 mm inosine (MCE, HY‐N0092), Rapamycin (MCE, HY‐10219), LW6 (MCE, HY‐13671), and PD98059 (MCE, HY‐12028) as described previously.

Cultures were established in 24‐well plates with 5 × 10^5^ cells/well in 500 µL of medium at 5% CO_2_ and 37 °C. Inosine (MCE, HY‐N0092) was added to the culture, while a blank control was set up without the addition of IL‐4 and R848 in the medium. After 48 h, all cells were harvested to evaluate B cell differentiation, and the supernatant was collected to detect antibody production using cytometric bead array. Furthermore, the impact of inosine signaling on transcription factors involved in B‐cell proliferation and differentiation was assessed using a western blot and qPCR.

### Western Blotting

Mouse splenic B cells were lysed with RIPA buffer (Beyotime, P0013K), and protein quantified with a BCA Protein Assay Kit (Beyotime, P0011). A 10% SDS‐page was loaded with the protein, which was then transferred onto polyvinylidene difluoride membranes (Millipore).

### RNA Extraction and qRT‐PCR

RNA was isolated from mouse spleen B cell using Total RNA Extraction Reagent (Vazyme, R401‐01‐AA). The RNA quality and concentration were determined using a NanoDrop spectrophotometer (Thermo, ND‐2000). cDNA was synthesized from the extracted RNA using the HiScript III RT SuperMix for qPCR (Vazyme, R323‐01) followed the manufacturer's procedures. qRT‐PCR using ChamQ Universal SYBR qPCR Master Mix (Vazyme, Q711‐02) was conducted on a Roche LightCycler 480 II instrument. The gene mRNA levels were normalized to the control group and calculated using the formula 2^^−ΔΔCt^ (where ΔΔCt = ΔCt of experimental group − ΔCt of control group).

### Statistics

Bars and error bars represent the mean and s.e.m., respectively. Non‐survival analysis was performed using Student's unpaired *t*‐test between two groups. ANOVA was used for comparisons with more than two groups. GraphPad Prism version 8.0 (GraphPad Software, San Diego, CA) was used for statistical treatment.

## Conflict of Interest

The authors declare no conflict of interest.

## Author Contributions

L.G., Y.Z., and Z.H. contributed equally to this work. Conceptualization was carried out by LQJ, FDY, ZM, and GLY. Methodology was dealt with by LQJ, FDY, ZM, GLY, HZ, CSW, YHQ, ZJP, and ZYJ. Investigation was performed by GLY, ZYH, CSW, CB, WQL, ZY, GCX, and XY. Visualization was carried out by LQJ, FDY, GLY, and HZ. Funding acquisition was performed by LQJ, ZM, and GLY. Project administration was carried out by LQJ, GLY, and ZM. Supervision was performed by LQJ. Writing–original draft was carried out by GLY. Writing–review & editing was performed by LQJ, FDY, ZM, and SVDV.

## Ethics Approval Statement

All human subjects were approved by the ethics committee of the Institute of Dermatology, Chinese Academy of Medical Sciences (2021.054). The animal ethical and care guidelines of the Institute of Dermatology, Chinese Academy of Medical Sciences (no. 2022‐DW‐017) were adhered to, and the approval by local government authorities was obtained.

## Supporting information



Supporting Information

Supporting Information

Supporting Information

## Data Availability

The data that support the findings of this study are available on request from the corresponding author. The data are not publicly available due to privacy or ethical restrictions.
