# Supplementary material for: Microbiota‐Derived Inosine Suppresses Systemic Autoimmunity via Restriction of B Cell Differentiation and Migration
Source: Adv Sci (Weinh). 2025 Apr 28;12(20):2409837. doi: 10.1002/advs.202409837 (PMC12120789; doi:10.1002/advs.202409837)
Supplement: Supplementary file 1 — Supporting Information [file ADVS-12-2409837-s001.pdf]

ADVANCED SCIENCE
Open Access

Supporting Information

for *Adv. Sci.*, DOI 10.1002/advs.202409837

Microbiota-Derived Inosine Suppresses Systemic Autoimmunity via Restriction of B Cell Differentiation and Migration

*Lingyu Gao, Yuhan Zhang, Zhi Hu, Shengwen Chen, Qiaolin Wang, Yong Zeng, Huiqi Yin, Junpeng Zhao, Yijing Zhan, Changxing Gao, Yue Xin, Bing Chen, Stijn van der Veen, Ming Zhao, Deyu Fang and Qianjin Lu**

Microbiota-derived Inosine Suppresses Systemic Autoimmunity via Restriction of B Cell Differentiation and Migration

Lingyu Gao[1, 2, 3], Yuhan Zhang[1, 2, 3], Zhi Hu [1, 2, 3], Shengwen Chen[1, 2, 3], Qiaolin Wang[1, 2, 3], Yong Zeng[1, 2, 3, 4], Huiqi Yin[1, 2, 3], Junpeng Zhao[1, 2, 3], Yijing Zhan[1,2,3], Changxing Gao[1, 2, 3], Yue Xin[1, 2, 3], Bing Chen[5], Stijn van der Veen[6], Ming Zhao[1, 2, 3, 4], Deyu Fang[7], Qianjin Lu[1, 2, 3, 4]*

L.G., Y.Z., and Z.H., contributed equally to this work.

*E-mail: qianlu5860@pumcderm.cams.cn

Supplementary figures and legends

Figs. S1 to S18

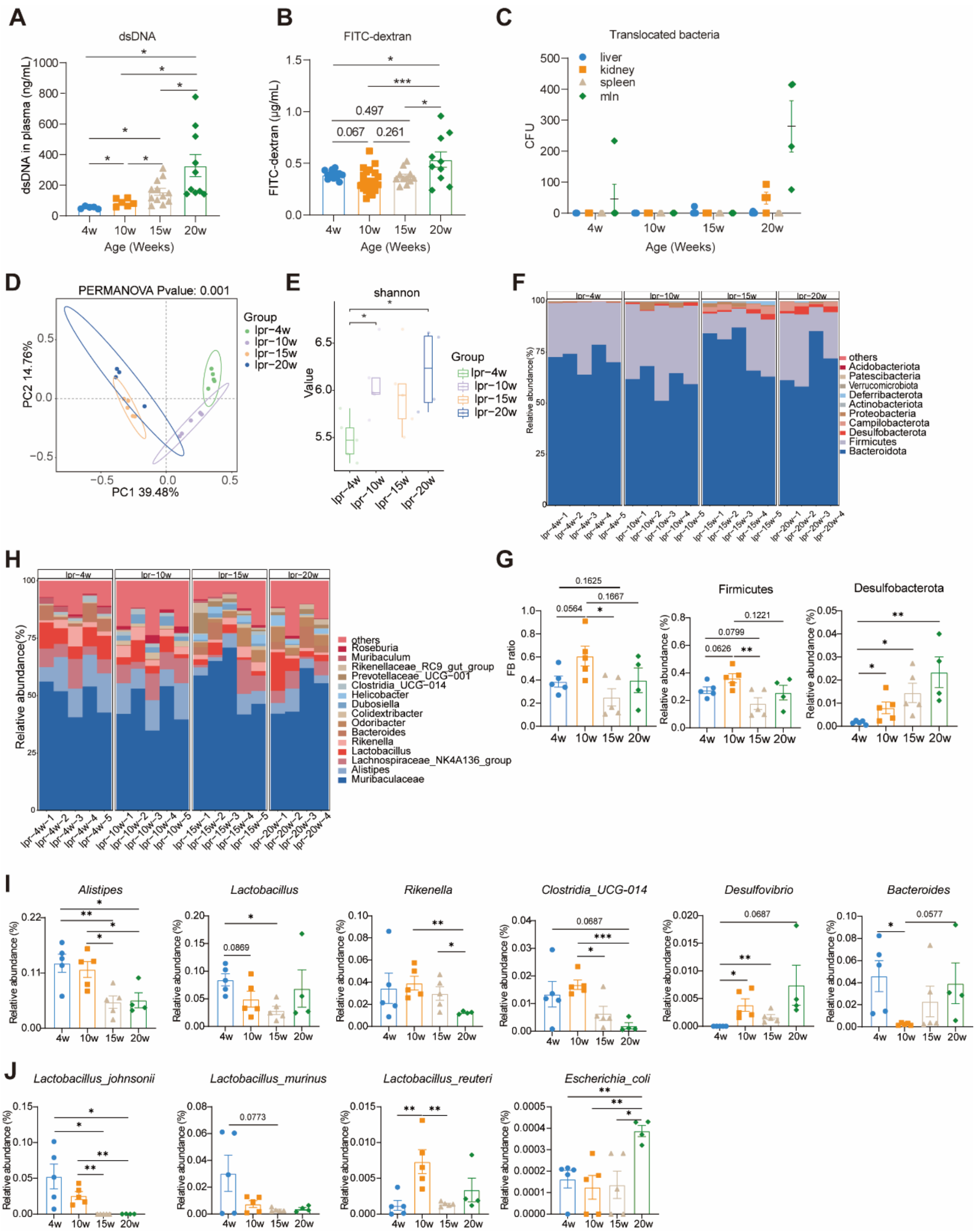

Fig. S1. Gut microbiota dynamics in spontaneous lupus-prone mice. (A) Plasma anti-dsDNA level from 4w, 10w, 15w, and 20w mice were detected (n=5-12 mice per group). **(B)** Plasma levels of orally fed FITC-dextran (4K) (n=10-22 mice per group). **(C)** Cultures of liver, kidney, spleen, and MLN tissues from MRL/lpr mice (n=6). **(D-I)** 16S rDNA sequencing of fecal pellets from 4w, 10w, 15w, and 20w mice (n=5 for 4w, 10w, 15w groups, n=4 for 20w group). (D) Beta-diversity and (E) alpha diversity among groups. (F and G) Relative abundance at phylum level and (H and I) genus level among groups. **(J)** Bacterial species abundance comparison among groups. The results are expressed as mean ± SEM (n=4-22 mice per group). Statistical comparison was based on an unpaired Student t test. *p<0.05 was considered statistically significant; **p<0.01; ***p<0.001.

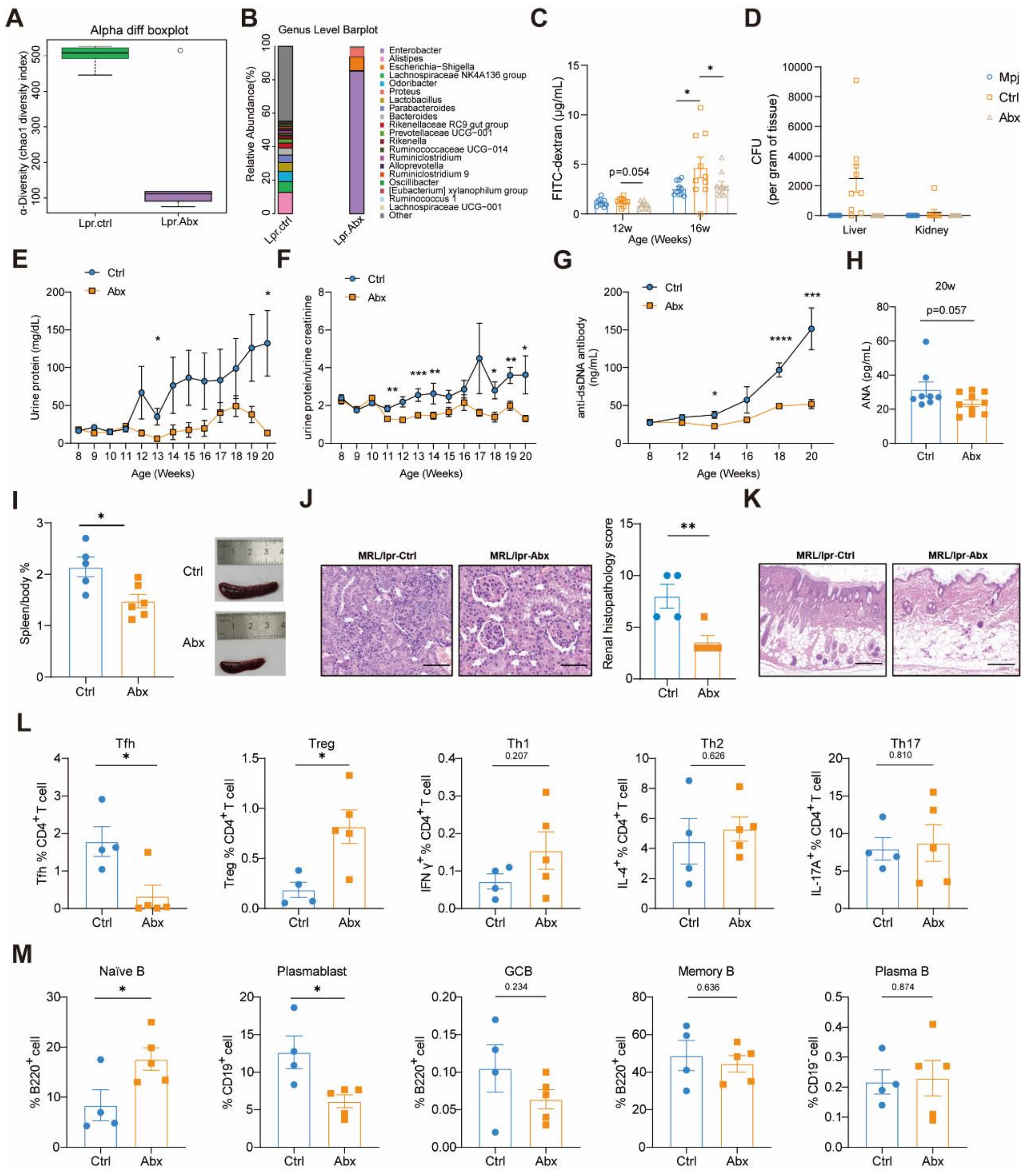

Fig. S2. 16S rDNA sequencing of fecal pellets from antibiotics- and control-treated MRL/lpr mice. A cocktail of antibiotics (Abx) containing vancomycin (0.25 g/l), ampicillin (0.5 g/l), metronidazole (0.5 g/l), neomycin (0.5 g/l) or control water (Ctrl) were orally administered in the drinking water starting at 8 weeks of age. Fecal pellets were analyzed at 20 weeks of age. **(A)** Plots of Chao1 diversity between the two groups. **(B)** Relative abundance of genus level. **(C)** Plasma FITC-dextran level in 12 and 16 weeks of age among Mpj, Ctrl and Abx groups. **(D)** Cultures of translocated bacteria in liver and kidney among Mpj, Ctrl and Abx groups in 20 weeks of age. **(E)** Urine protein was detected. **(F)** The ration of urine protein and creatinine was detected. **(G)** Plasma anti-dsDNA level was detected between Ctrl and Abx groups (n>=8 per group). **(H)** Plasma ANA level in 20 weeks of age (n>=8 per group). **(I)** The ratio of spleen weight to body weight in 20 weeks of age (n>=5 per group). **(J and K)** Renal histopathology (J) and skin lesion (K) at 20 weeks of age ($n \geq 5$ per group), scale bar, 200 μm. **(L)** Frequencies of Tfh cells, Treg cells, Th1 cells, Th2 cells, and Th17 cells in spleen between Ctrl and Abx groups (n>=4 per group). **(M)** Frequencies of naïve

B cells, memory B cells, GCB cells, plasmablast, plasma cells in spleen between Ctrl and Abx groups (n>=4 per group). The results are expressed as mean ± SEM. Statistical comparison was based on an unpaired Student t test. *p<0.05 was considered statistically significant; **p<0.01; ***p<0.001; ****p<0.0001.

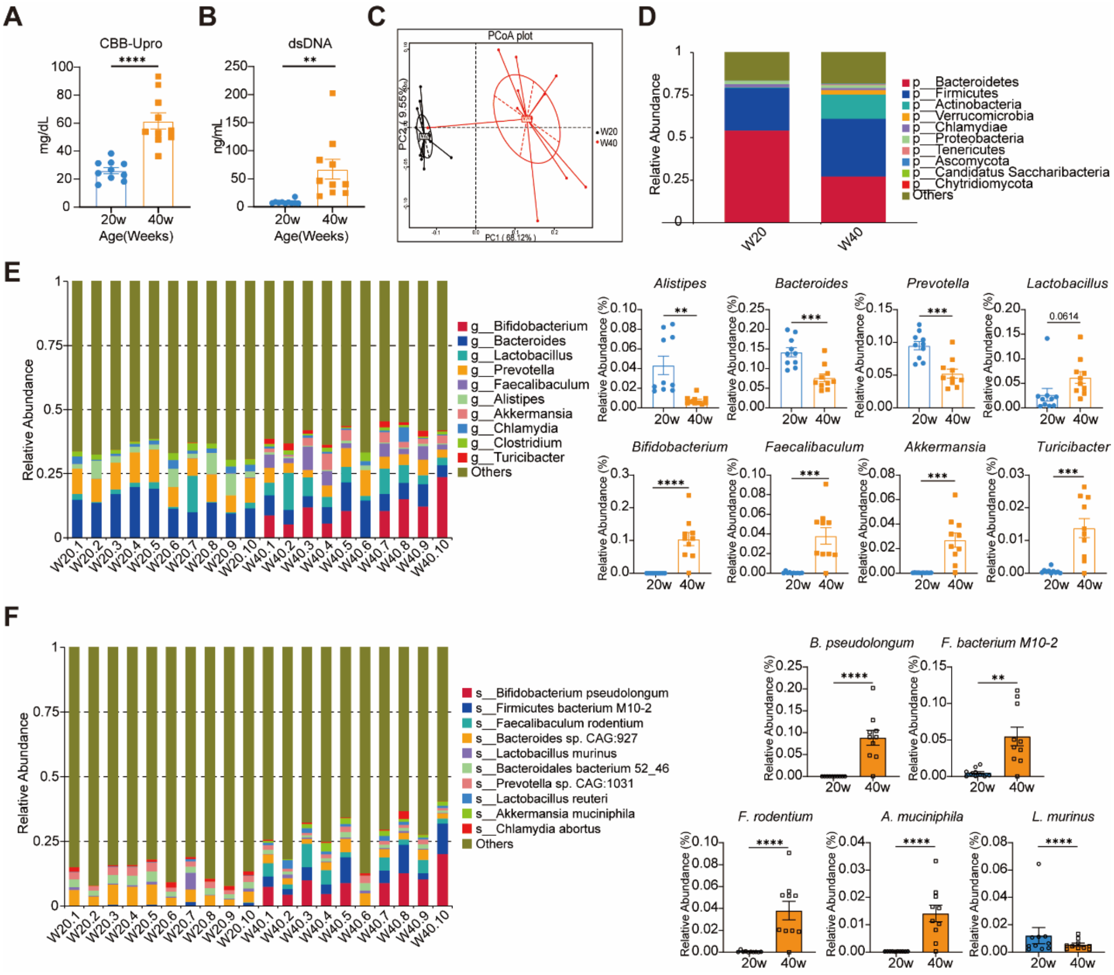

Fig. S3. Gut microbiota dynamics in pristane-induced lupus mice. Fecal metagenomic analysis between 20 weeks and 40 weeks of age in pristane-induced C57BL/6j mice. **(A)** Urine protein was detected by CBB method. **(B)** Plasma anti-dsDNA level was detected between 20 weeks and 40 weeks of age (n=10 per group). **(C)** Beta diversity analyses of Bray–Curtis similarity index scores of microbial species between 20 weeks and 40 weeks of age (n=10 per group). **(D** to **F)** Relative abundance at phylum level (D), genus level (E) and species level (F). The results are expressed as mean ± SEM (n=10 mice per group). Statistical comparison was based on an unpaired Student t test. *p<0.05 was considered statistically significant; **p<0.01; ***p<0.001; ****p<0.0001.

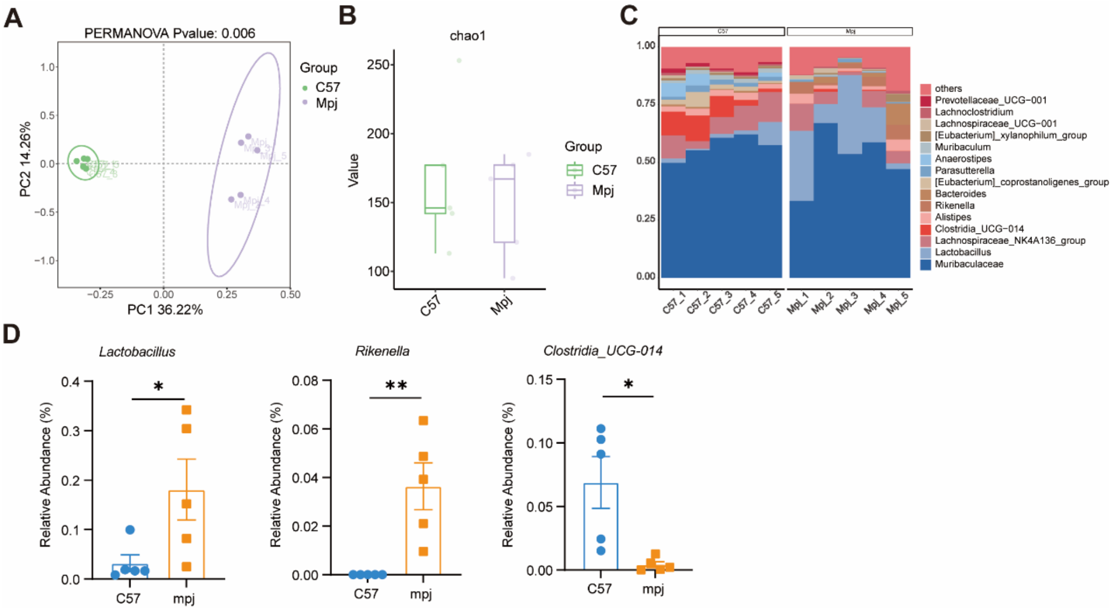

Fig. S4. 16S rDNA sequencing of fecal pellets from C56BL/6j and MRL/mpj mice. Fecal pellets collected from 8 weeks of age C56BL/6j and age-matched MRL/mpj mice (n=5 per group). **(A** and **B)** Beta-diversity (A) and alpha diversity (B) between these two groups. **(C** and **D)** Relative abundance of the top 15 genera (C) and relative abundance of genus *Lactobacillus*, *Rikenella*, and *Clostridia_UCG-014* (D). The results are expressed as mean ± SEM. Statistical comparison was based on an unpaired Student t test. *p<0.05 was considered statistically significant; **p<0.01.

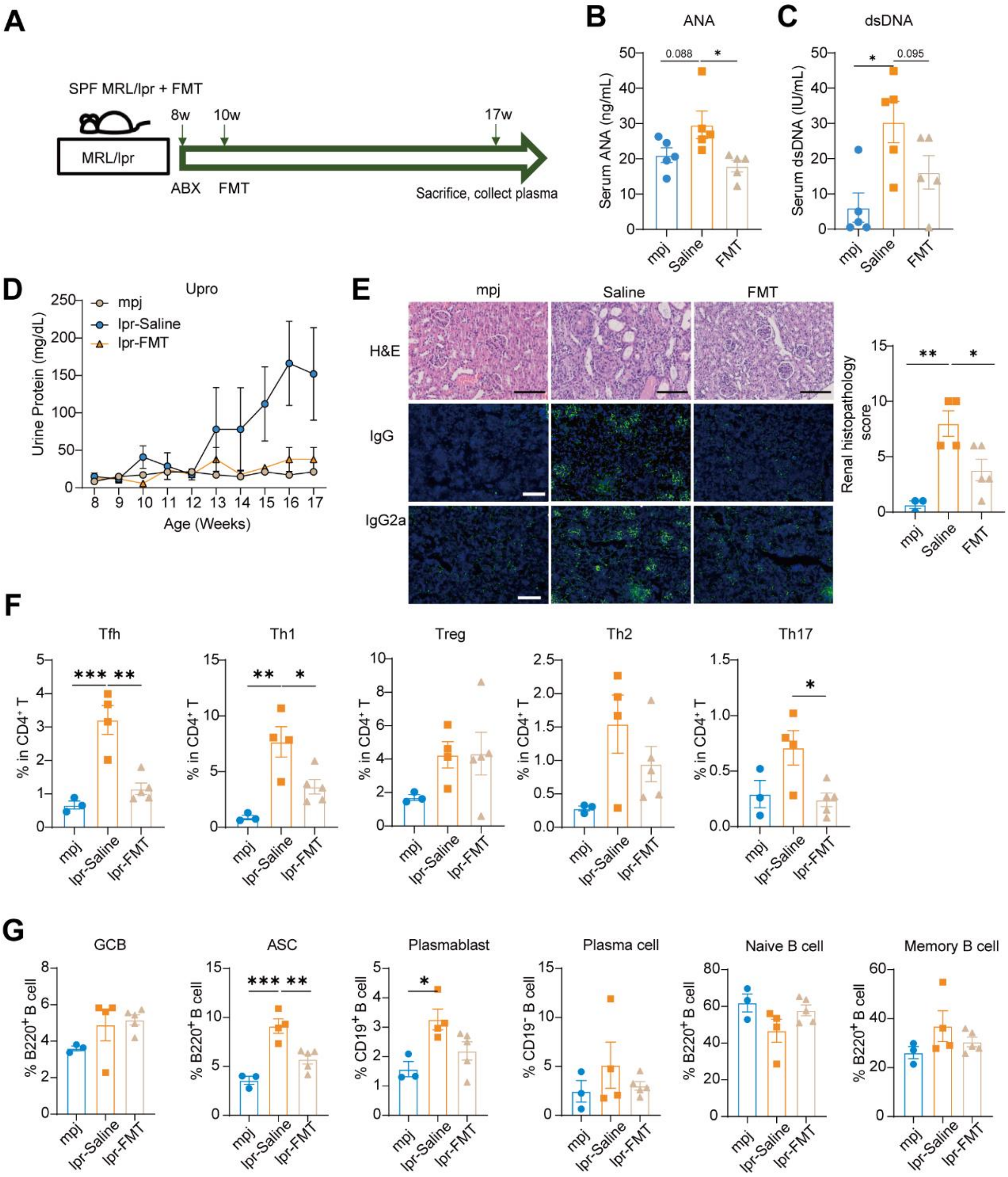

Fig. S5. Transplantation of fecal microbiota from MRL/mpj mice attenuated lupus-like phenotypes in MRL/lpr mice. (A) Overview of FMT experimental design (n=5 per group). 8-week age lpr mice were treated with a cocktail of antibiotics in drinking water for 2 weeks, and FMT (0.1g/mL, 0.2mL/time, 3 times/week) or saline treatment started at 10 weeks of age and lasted until 17 weeks of age. **(B** and **C)** Plasma ANA level (B) and anti-dsDNA level (C) between saline-treated and FMT-treated groups. **(D)** Urine protein was detected by test strip. **(E)** Renal histopathology, IgG, IgG2a deposition in the kidney were examined. Scale bar of H&E, 100μM; IgG and IgG2a, 200μM. **(F)** Frequencies of Tfh cells, Th1 cells, Treg cells, Th2 cells, and Th17 cells in spleen between saline-treated and FMT-treated groups. **(G)** Frequencies of GCB cells, ASC cells, plasmablast cells, plasma cells, naïve B cells and memory B cells in spleen between saline-treated and FMT-treated groups. The results are expressed as mean ± SEM. Statistical comparison was based on an unpaired Student t test. *p<0.05 was considered statistically significant; **p<0.01,

***p<0.001.

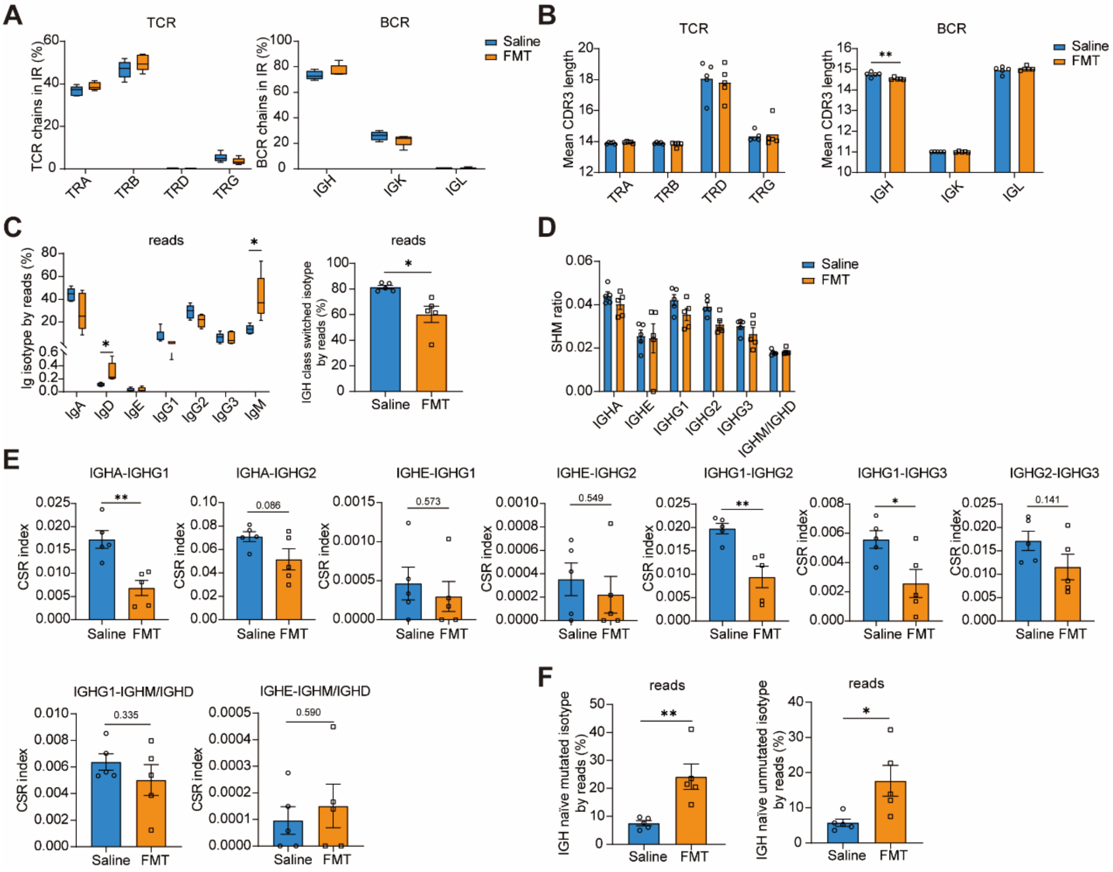

Fig. S6. Related to Figure 2. Overall analysis of the composition of BCR and TCR in spleen from pristane-induced mice with or without FMT treatment. (A) Percentage of seven chains (4 in TCR, 3 in BCR) by counting reads in the spleen from pristane-induced mice of the saline and FMT groups **(B)** Specific statistical analysis of each BCR and TCR chains mean length between saline and FMT groups. **(C)** Expression percentage calculated in each IGH chain isotype and IGH class switched isotype percent by reads from group saline and FMT. **(D)** Somatic hypermutation (SHM) ratio in six Ig subtypes. **(E)** The CSR index of IGHA with IGHG1, IGHG2, and IGHE with IGHG1, IGHG2, and IGHG1 with IGHG2, IGHG3, IGHG2 with IGHG3, IGHG1 with IGHM/IGHD, IGHE with IGHM/IGHD in the two groups. **(F)** IGH naïve mutated isotype percentage and IGH naïve mutated isotype percentage by reads. Data were obtained from five biologically replicated experiments. The results are expressed as mean ± SEM. Statistical comparison was based on an unpaired Student t test. *p<0.05 was considered statistically significant; **p<0.01.

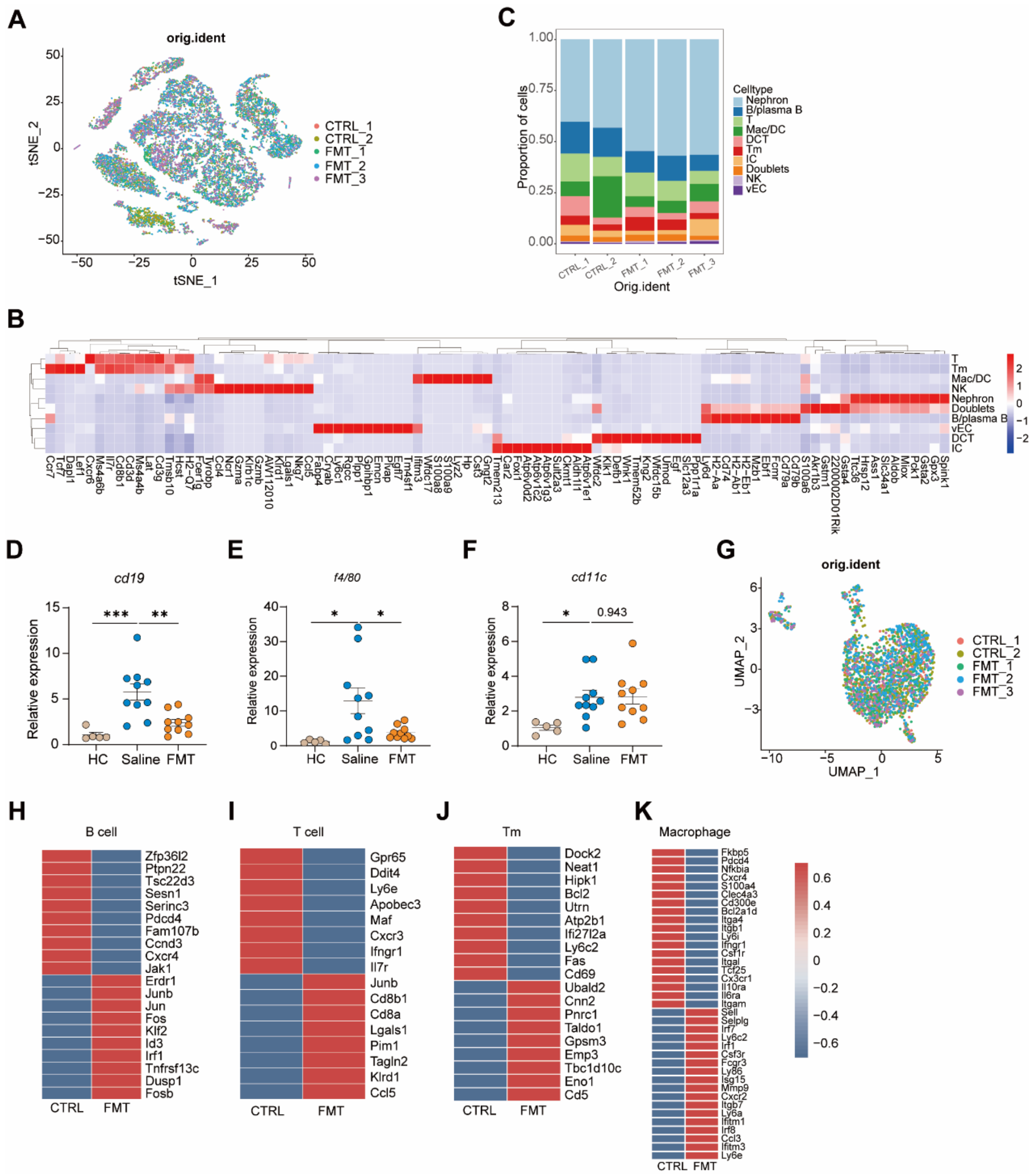

Fig. S7. (Related to Figure 3) Gut microbiota alteration reduced immune cell infiltration in mice kidneys. (A) scRNA-seq t-SNE plot showing renal cells colored by individual samples. (FMT: FMT-treated lupus mice, CTRL: saline-treated control). **(B)** Heatmap plot showing the average expression of the top 10 differential genes for each cell type. The color represents the scaled amount of expression for each gene across cell types. **(C)** Bar plot showing the abundance of cell types for each sample. **(D-F)** The mRNA level of *cd38, f4/80,* and *cd11c* in kidney. **(G)** scRNA-seq Umap plot showing renal B cells colored by individual samples. **(H-K)** Heatmap plot showing differential genes of B cell (H), T cell (I), memory T cell (J), Macrophage and Dendritic cell (K), for CTRL and FMT. The color represents the scaled amount of expression across groups. The results are expressed as mean ± SEM. Statistical comparison was based on one-way ANOVA. *p<0.05 was considered statistically significant; **p<0.01; ***p<0.001; ****p<0.0001.

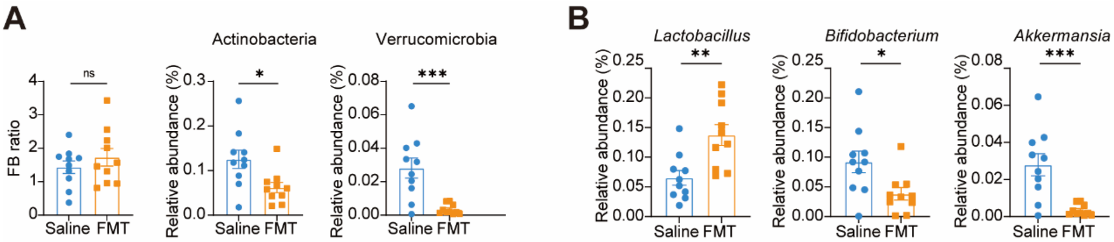

Fig. S8. Related to Figure 4. Changes in gut microbiota after FMT in pristane-induced mice. (A) The F/B ratio, phylum *Actinobacteria* and *Verrucoicrobia* were analyzed. (B) Relative abundance of genus *Lactobacillus, Bifidobacterium and Akkermansia* between saline-treated and FMT-treated groups. The results are expressed as mean ± SEM. Statistical comparison was based on an unpaired Student t test. *p<0.05 was considered statistically significant; **p<0.01; ***p<0.001.

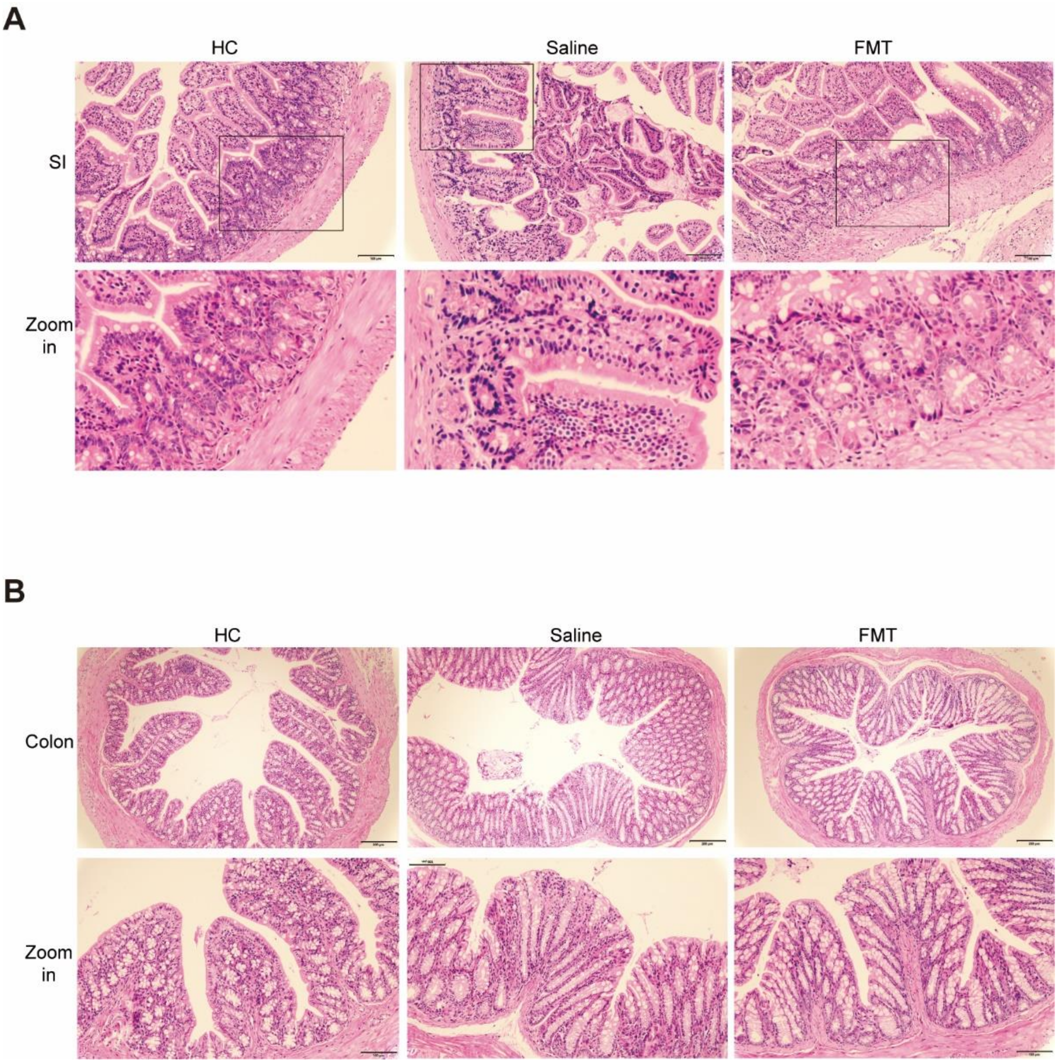

Fig. S9. Effect of FMT on gut pathology in pristane-induced mice. (A) Histopathologic changes on H&E-stained slides prepared from small intestines. **(B)** Histopathologic changes on H&E-stained slides prepared from colon. Scale bar: 200μm.

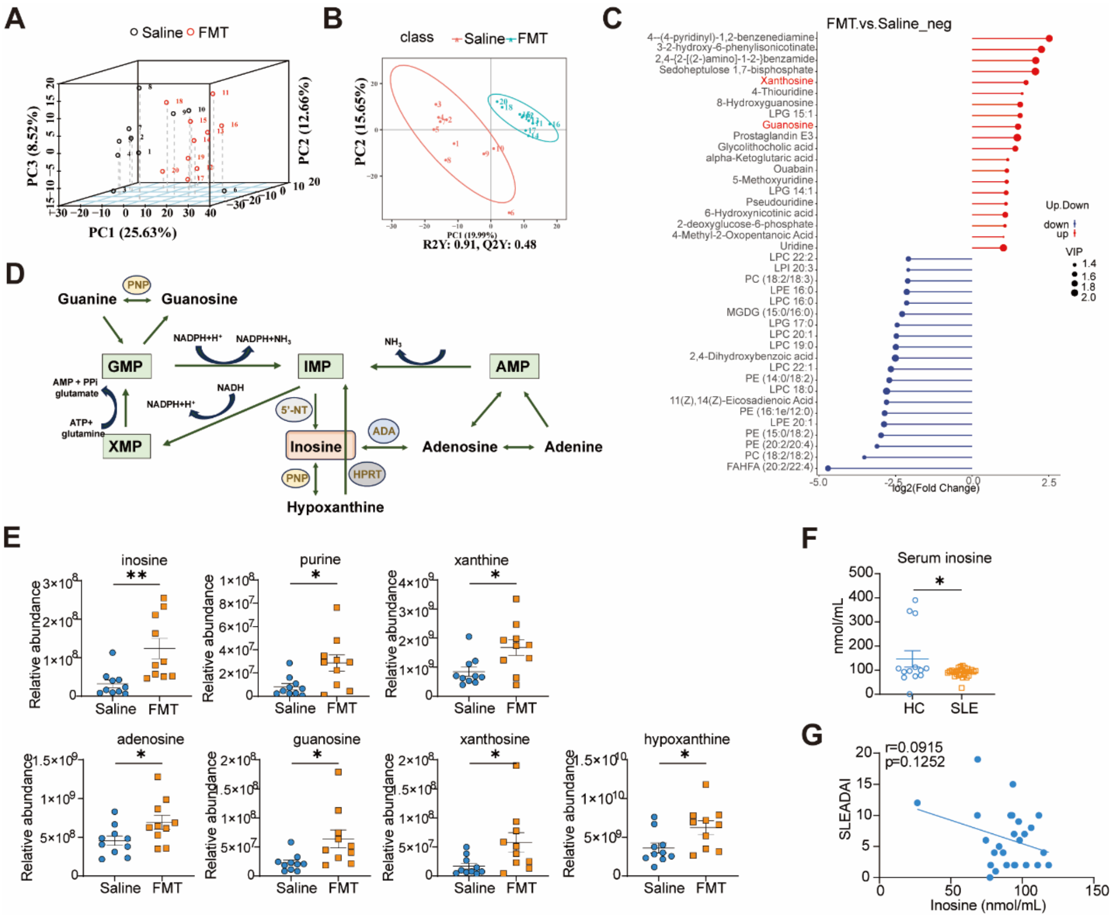

Fig. S10 Related to Figure 4. FMT modulated fecal metabolites profiles and increased purine metabolism. An untargeted metabolomics of the feces in saline-treated and FMT-treated pristane-induced mice was conducted (n=10 per group). **(A** and **B)** Three-dimension image of principal component analysis (PCA) (A) and Partial Least Squares Discrimination Analysis (PLS-DA) (B) in positive mode between these two groups. **(C)** The matchstick map according to the differential metabolites obtained from each group of difference comparison combinations in negative mode was drawn, and the up-down of metabolites and the substances with large difference multiples were indicated. **(D)** Schematic depiction of purine metabolism. HPRT, hypoxanthine guanine phosphoribosyl transferase; ADA, adenosine deaminase; PNP, purine nucleoside phosphorylase; 5'-NT, 5' Nucleotidase. **(E)** Relative abundance of fecal purine metabolites in saline-treated and FMT-treated pristane-induced mice. **(F)** The amount of inosine in serum samples from healthy control (HC, n=14) and SLE patients (SLE, n=28). **(G)** Correlation analysis of the amount of inosine with SLEADAI scores. The correlation analysis was used linear regression. Statistical comparison was based on an unpaired Student t-test. **p*<0.05 was considered statistically significant, ***p*<0.01.

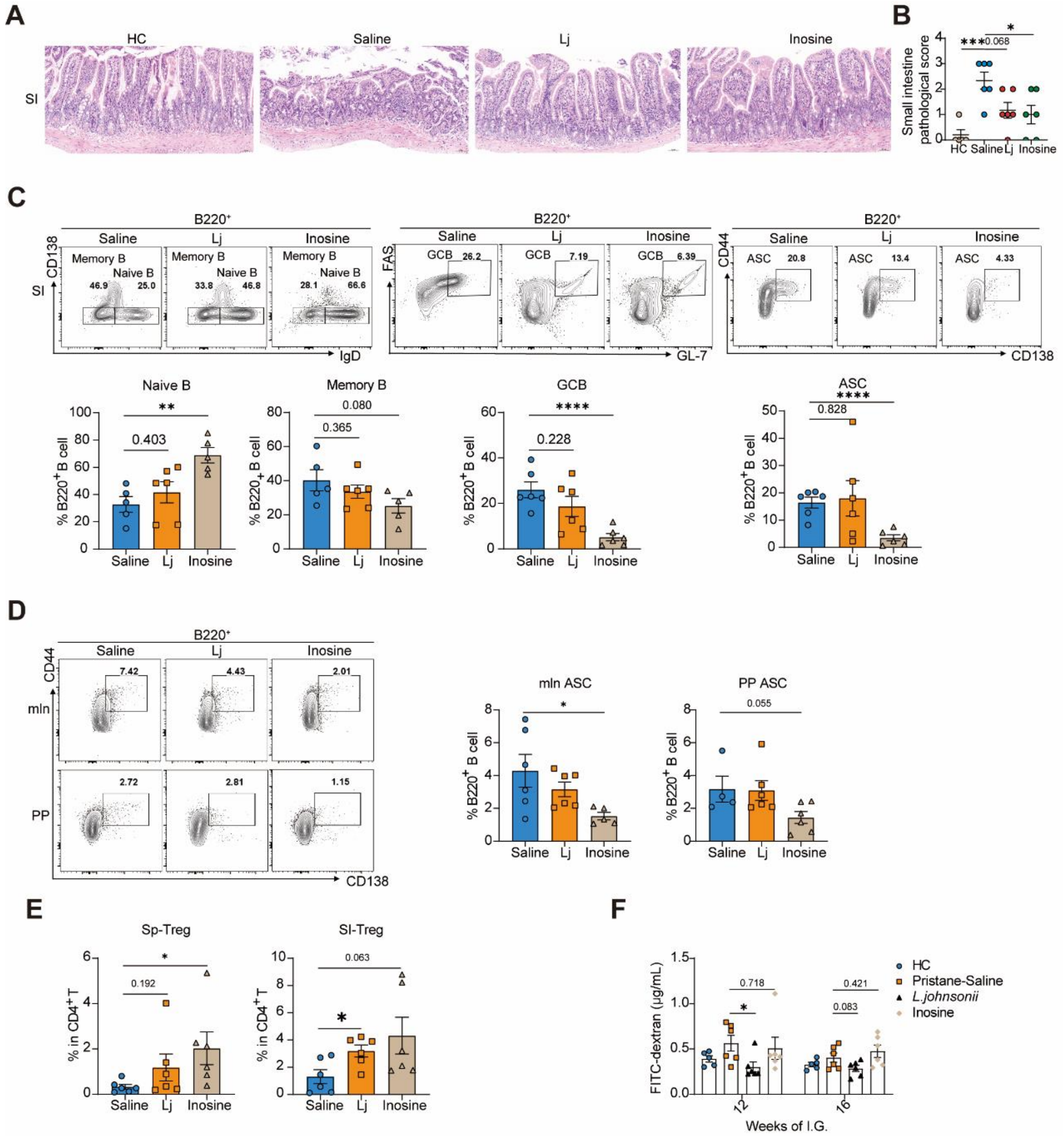

Fig. S11 Related to Figure 5. *L. johnsonii* and inosine treatment ameliorated lupus-like symptoms in lupus mice. (A) Histopathologic changes on H&E-stained slides prepared from small intestines. Scale bar: 50μm. **(B)** Small intestines pathological score in (A). **(C)** Statistical analysis of frequencies of naïve B cells, memory B cells, GCB cells and ASC in small intestines among saline-treated, Lj-treated, and inosine-treated groups. **(D)** Statistical analysis of frequencies of ASC in MLN and PP among saline-treated, Lj-treated, and inosine-treated groups (n=6 per group). **(E)** Statistical analysis of frequencies of Treg cells in spleen and small intestines among saline-treated, Lj-treated, and inosine-treated groups (n=6 per group). **(F)** Plasma FITC-dextran level in 12-week and 16-week treatment among healthy control (n=5), saline-treated, Lj-treated, and inosine-treated groups (n=6 per group). The results are expressed as mean ± SEM. Statistical comparison was based on an unpaired Student t-test. *p<0.05 was considered statistically significant; **p<0.01.

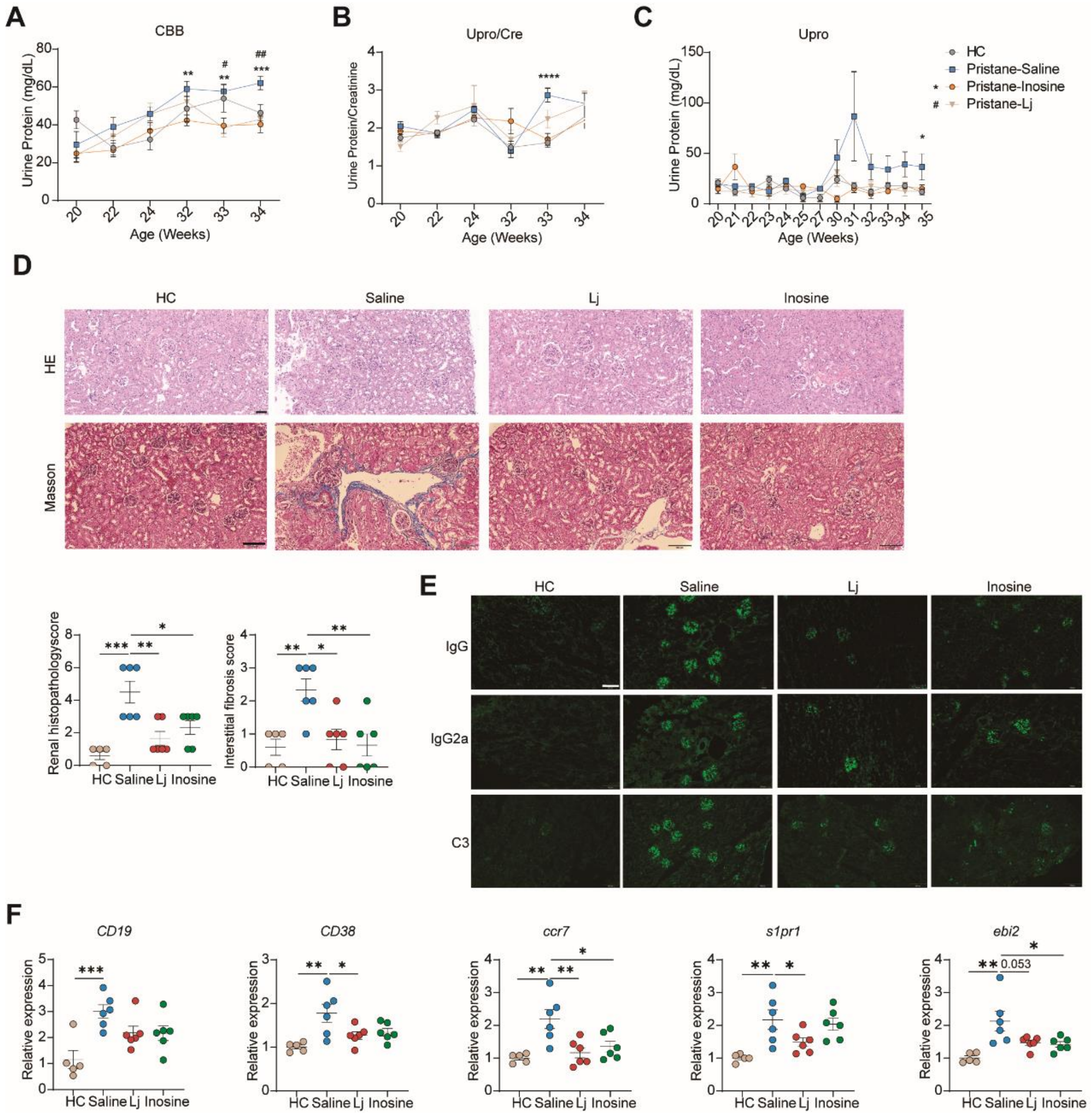

Fig. S12. ***L. johnsonii*** **and inosine treatment ameliorated lupus nephritis in pristane-induced mice. (A)** Urine protein was detected by the CBB method among saline-treated, Lj-treated, and inosine-treated groups. **(B)** The ratio of urine protein and creatinine was detected. **(C)** Urine protein was detected by the test strip. **(D)** H&E and Masson staining of the kidney, renal histopathology score, and interstitial fibrosis score were evaluated. **(E)** IgG, IgG2a, and C3 deposition in the kidney were detected. Scale bar, 200μm. **(F)** Relative expression of *cd19, cd38, ccr7, s1pr1* and *ebi2* mRNA were quantified. The results are expressed as mean ± SEM. Statistical comparison was based on one-way ANOVA. *p<0.05 was considered statistically significant; **p<0.01; ***p<0.001; ****p<0.0001.

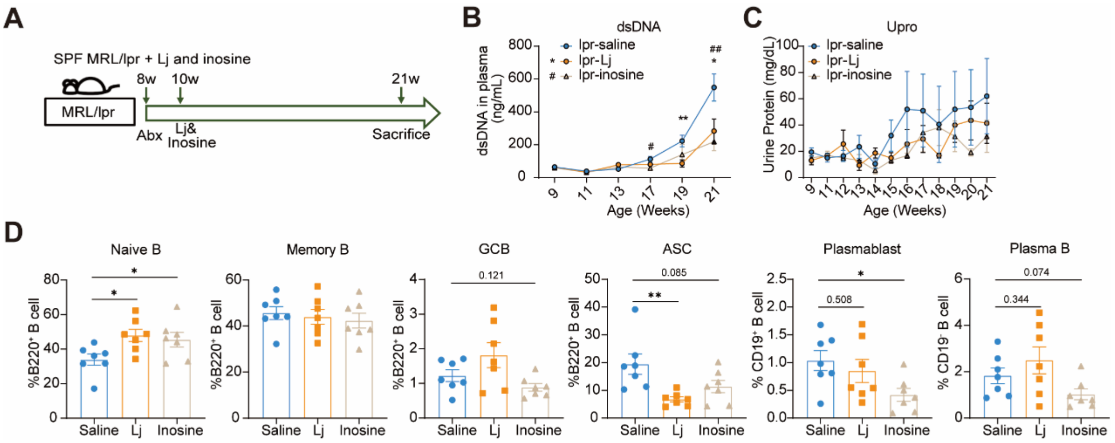

Fig. S13. *L. johnsonii* and inosine treatment ameliorated lupus-like symptoms in MRL/lpr mice. (A) Overview of *L. johnsonii* (Lj) and inosine treatment experimental design (n=7 per group). Before orally gavage Lj and inosine, antibiotics were fed to lpr mice for two weeks. Lj (2 OD, 0.3mL/time, 3 times/week), inosine (50mg/kg, 0.3mL/time, 3 times/week), or saline treatment started at 10 weeks and lasted until 21 weeks. **(B)** Anti-dsDNA level among saline-treated, Lj-treated, and inosine-treated groups. **(C)** Urine protein was detected by the test strip. **(D)** Statistical analysis of frequencies of naive B cells, memory B cells, GC B cells, ASC, plasmablast, and plasma cells in spleen among saline-treated, Lj-treated, and inosine-treated groups. The results are expressed as mean ± SEM. Statistical comparison was based on an unpaired Student t-test. *p<0.05 was considered statistically significant; **p<0.01.

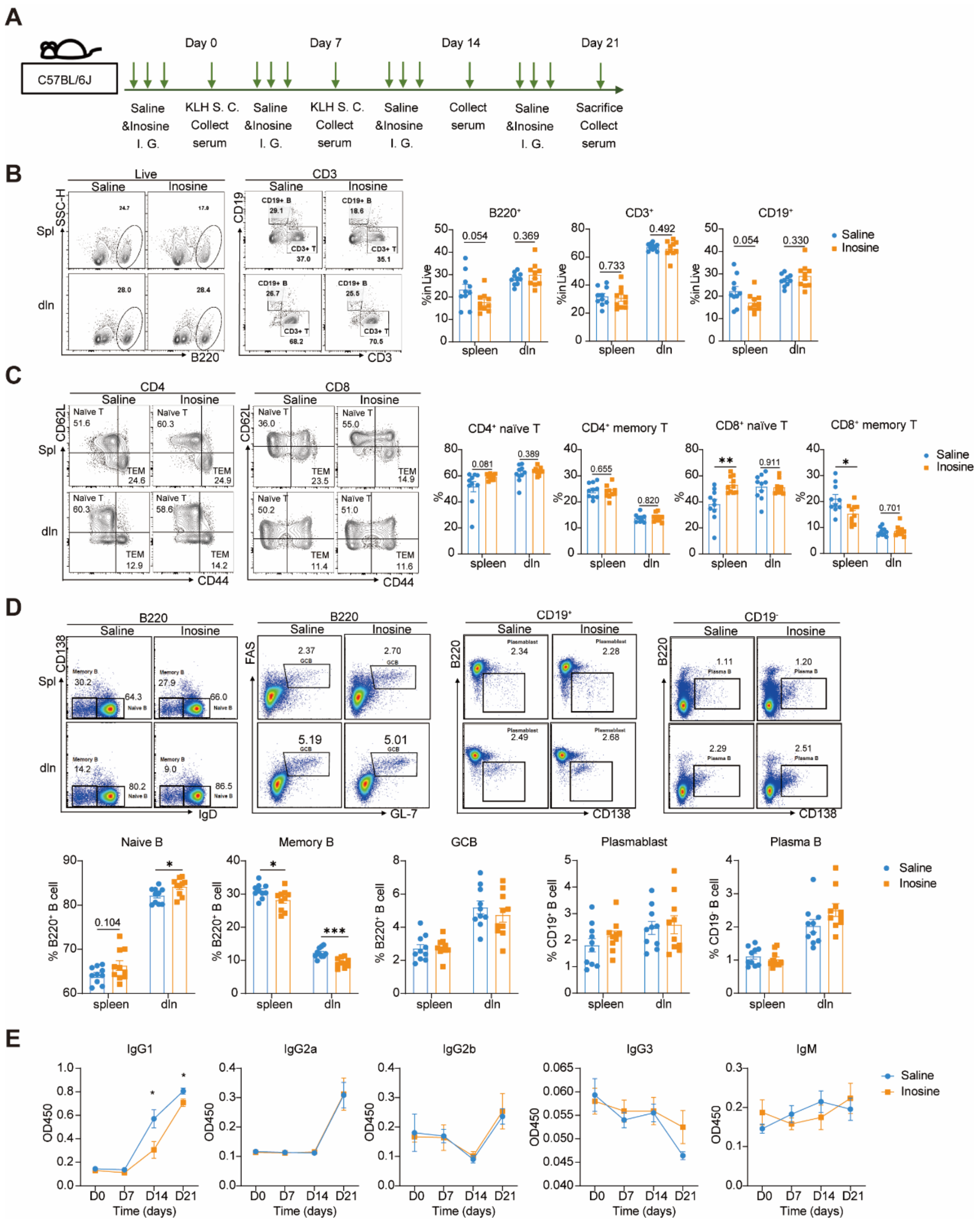

Fig. S14. The KLH-induced response in saline-treated and inosine-treated mice. (A) Overview of saline and inosine treatment experimental design (n=10 per group). C57BL/6j mice were subcutaneously injected 150uL KLH at 9 and 10 weeks. Inosine (50mg/kg, 0.3mL/time, 3 times/week) or saline treatment lasted 21 days. **(B)** Representative flow cytometry diagrams and statistical analysis of frequencies of B220 cells, CD3 cells, and CD19 cells in spleen and draining lymph nodes between saline-treated and inosine-treated groups. **(C)** Representative flow cytometry diagrams and statistical analysis of frequencies of CD4 naïve T cells, CD4 TEM cells, CD8 naïve T cells, and CD8 TEM cells in spleen and draining lymph nodes between saline-treated and inosine-treated groups. **(D)** Representative flow cytometry diagrams and statistical analysis of frequencies of naive B cells, memory B cells, GC B cells, plasmablast, and plasma cells in spleen and draining

lymph nodes between saline-treated and inosine-treated groups. **(E)** The serum levels of total IgG1, IgG2a, IgG2b, IgG3, and IgM from the KLH-immunized mice on days 0, day 7, day 14, day 21. The results are expressed as mean ± SEM. Statistical comparison was based on an unpaired Student t-test. *p<0.05 was considered statistically significant; **p<0.01; ***p<0.001.

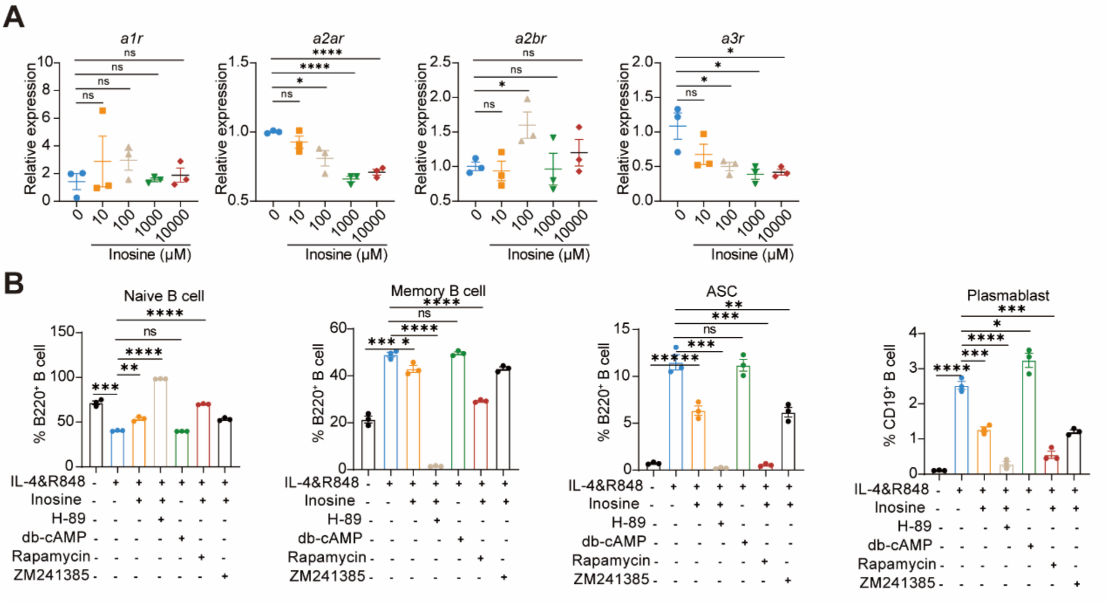

Fig. S15. Inosine restricting B cell differentiation was not via the A2aR-cAMP-PKA pathway. (A) The mRNA level of inosine receptors A1R, A2aR, A2bR, and A3 with different concentrations of inosine (0, 10uM, 100uM, 1mM, 10mM) for 48 hours. **(B)** Statistical analysis of frequencies of naive B cells, memory B cells, ASC, and plasmablast with inosine (1mM) for 48 hours together treated with PKA inhibitor H-89, cAMP analog db-cAMP, mTOC1 inhibitor rapamycin, and A2aR inhibitor ZM241385. Data were obtained from three biologically replicated experiments. The results are expressed as mean ± SEM. Statistical comparison was based on an unpaired Student t-test. *p<0.05 was considered statistically significant; **p<0.01; ***p<0.001; ****p<0.0001; ns = not significant.

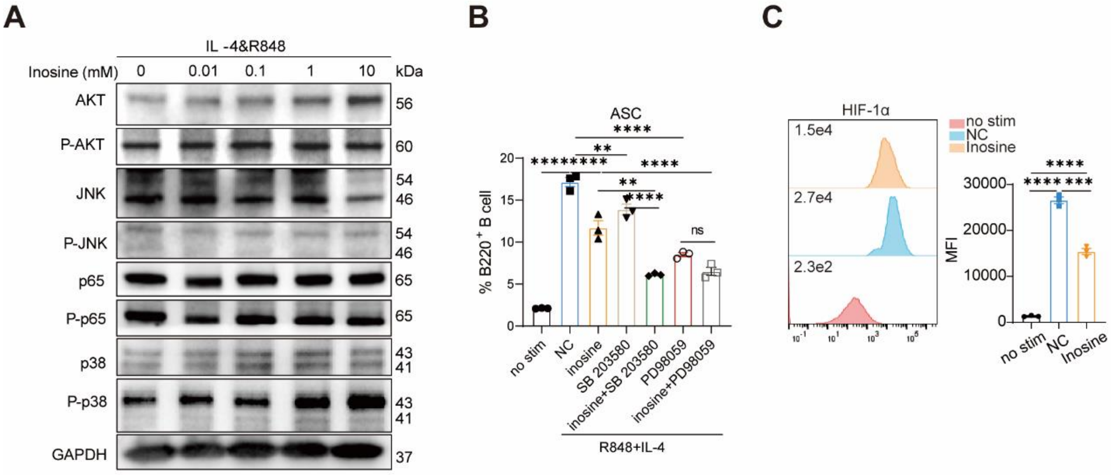

Fig. S16. Signaling pathways activation after increased inosine treatment. (A) Western blot detected MAPK (p38 MAPK and JNK), AKT, and P65 activation after inosine treatment. **(B)**

Statistical analysis of frequencies of ASC with or without p38 MAPK inhibitor, SB 203580 (10μM) in human peripheral blood B cells culture for 48h. (C) The fluorescence level of HIF-1α in human peripheral blood B cells was incubated with inosine (1mM) for 48h. Human peripheral blood B cells were isolated using magnetic beads and subsequently activated with IL-2 and R848 for 48 hours. The results are expressed as mean ± SEM. Statistical comparison was based on an unpaired Student t-test. **p*<0.05 was considered statistically significant; ***p*<0.01; ****p*<0.001; *****p*<0.0001.

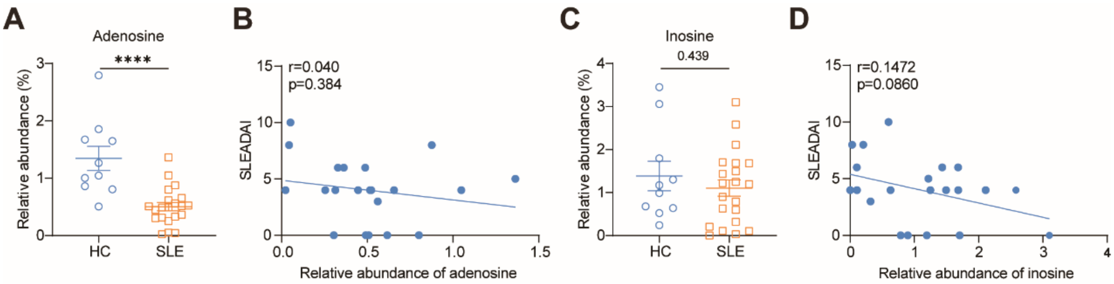

Fig. S17. Data analysis from *He et al., 2020* of relative abundance of adenosine and inosine in feces and its correlation with SLEDAI scores. (A) Relative abundance of adenosine in HC and SLE patients. **(B)** The correlation analysis of adenosine level with SLEDAI scores. **(C)** Relative abundance of inosine in HC and SLE patients. **(D)** The correlation analysis of inosine level with SLEDAI scores. The correlation analysis was used linear regression. The results are expressed as mean ± SEM. Statistical comparison was based on an unpaired Student t-test. **p*<0.05 was considered statistically significant; *****p*<0.0001.

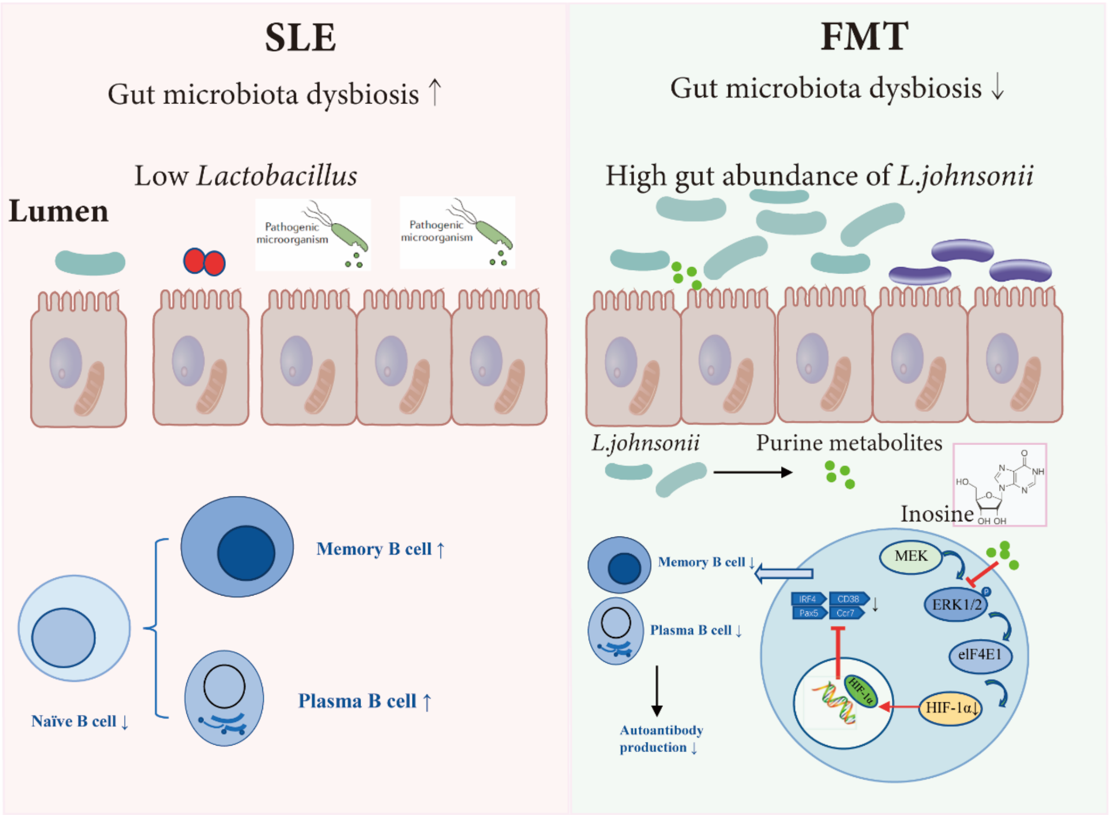

Fig. S18. Mechanism of microbiota-mediated autoimmunity alleviation. SLE patients and mice models have gut microbiota dysbiosis. Increasing the intestinal abundance of *L.johnsonii* resulted

in an increase in the purine metabolite inosine. Inosine reduced the phosphorylation of ERK1/2, thus modulating the downstream HIF1α expression, leading to the restriction of B cell differentiation and migration.
